# Antibody-trapping presents a widespread pitfall for microscopy and genomics in the nucleus

**DOI:** 10.1093/nar/gkag615

**Published:** 2026-06-27

**Authors:** Konrad Chudzik, Yuko Sato, Xingchi Yan, Simon Ullrich, Watanya Trakarnphornsombat, Lothar Schermelleh, Geoffrey Fudenberg, Hiroshi Kimura, Michael I Robson, Irina Solovei

**Affiliations:** Max-Delbrück-Center for Molecular Medicine in the Helmholtz Association (MDC), Berlin Institute for Medical Systems Biology (BIMSB), Chromatin (dys)function in disease group, Berlin, 10115, Germany; RG Development & Disease, Max Planck Institute for Molecular Genetics, Berlin, 14195, Germany; Cell Biology Center, Institute of Innovative Research, Tokyo Institute of Technology, Yokohama, 226-8501, Japan; School of Life Science and Technology, Tokyo Institute of Technology, Yokohama, 226-8501, Japan; Department of Quantitative and Computational Biology, University of Southern California, Los Angeles, CA 90089, United States; Faculty of Biology, Ludwig Maximilians University Munich, Planegg-Martinsried, 82152, Germany; School of Life Science and Technology, Tokyo Institute of Technology, Yokohama, 226-8501, Japan; Department of Biochemistry, University of Oxford, Oxford, OX1 3QU, United Kingdom; Department of Quantitative and Computational Biology, University of Southern California, Los Angeles, CA 90089, United States; Cell Biology Center, Institute of Innovative Research, Tokyo Institute of Technology, Yokohama, 226-8501, Japan; School of Life Science and Technology, Tokyo Institute of Technology, Yokohama, 226-8501, Japan; Cell Biology Center, Institute of Integrated Research, Institute of Science Tokyo, Yokohama, 226-8501, Japan; Max-Delbrück-Center for Molecular Medicine in the Helmholtz Association (MDC), Berlin Institute for Medical Systems Biology (BIMSB), Chromatin (dys)function in disease group, Berlin, 10115, Germany; Faculty of Biology, Ludwig Maximilians University Munich, Planegg-Martinsried, 82152, Germany

## Abstract

Chromatin has a complex 3D structure and diverse binding proteins that coordinate the genome’s most essential functions. Many microscopy and genomics technologies that map chromatin proteins and modifications rely on the diffusion of antibodies (Abs) to target epitopes within whole nuclei. Here, we reveal a critical flaw in such methods that arises when Abs become trapped at the edge of nuclear structures and fail to reach internally positioned epitopes. This “Ab-trapping” results in artifactual peripheral signal that fundamentally distorts the apparent positions of chromatin features across the genome and nucleus. Using computational modeling and experimental validation, we demonstrate that Ab-trapping is caused by a combination of three compounding factors—high epitope abundance, high Ab affinity, and low Ab diffusion rates. Ab-trapping can thus systematically misrepresent the localization of many prevalent chromatin features like histone modifications, transcription factors, nucleolar proteins, and protein tags. We also show that this artifact manifests in multiple technologies, including immunofluorescence microscopy, more recent CUT&Tag-seq, and likely any method relying on Ab diffusion. Finally, we outline readily implementable strategies to identify and mitigate Ab-trapping. Combined, our work presents a previously unrecognized yet prevalent artifact in Ab-based chromatin mapping methods and the means to resolve it.

## Graphical Abstract

**Graphical Abstract:**
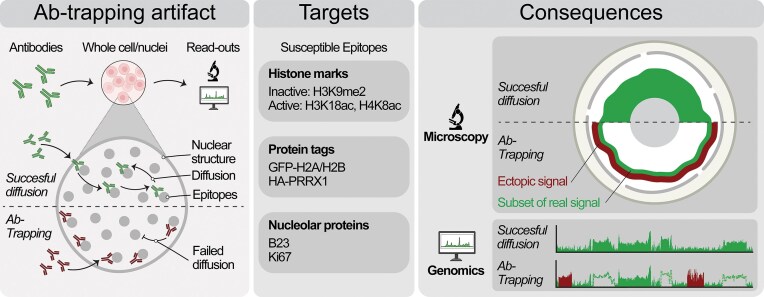

## Introduction

Chromatin has an elaborate composition and 3D structure that regulates many key genomic processes, including transcription, DNA replication, ribosome biogenesis, and DNA repair [1–3]. For example, the distribution of transcription factors and post-translational histone modifications throughout the genome defines the active or repressed epigenetic states of genes [4, 5]. Similarly, loci with distinct epigenetic states, DNA replication timing, and DNA repair events all display distinct spatial positions in the nucleus that are essential for proper function. The nuclear lamina and nucleoli physically associate with heterochromatin and are linked to transcriptional repression, late DNA replication, and homology-directed DNA repair [1, 6, 7]. Conversely, the euchromatic nuclear interior supports active transcription near nuclear speckles, early DNA replication, and non-homologous end joining-directed DNA repair [8, 9]. Due to these relationships, accurate mapping of protein distribution along chromatin and in the nucleus is key for understanding how the genome functions or is disrupted in disease [10, 11]. By extension, experimental artifacts that mischaracterize these distributions are therefore major impediments to research into genome biology in health and disease.

Numerous technologies have been developed to map chromatin’s epigenetic state and spatial organization, including many that rely on Abs diffusing to target epitopes within nuclei, cells, or tissues [12, 13]. Conjugated Abs are widely used to map the localization and abundance of proteins in fixed cells by immunofluorescence microscopy and, more recently, spatial proteomics methods [14–17]. Similarly, sequencing-based methods also employ Abs in whole nuclei for genome-wide chromatin feature mapping, including CUT&RUN/ChIC, CUT&Tag, pA-DamID, and MAbID [18–22]. These proven technologies have collectively become essential tools for uncovering the key principles of genome regulation enumerated above. What’s more, these methods underlie emerging technologies to map epitopes in single-cells [20, 22–24] and tissues [25], and so are set to impact research for the foreseeable future.

Though essential, Ab-based methods are subject to well-known artifacts that must be avoided. This includes Ab cross-reactivity to undesirable targets, epitope masking, or fixation-induced changes in protein distribution [26–28]. However, there are also differences in reported Ab staining patterns that cannot be explained by these known artifacts, posing significant challenges to our understanding of chromatin biology. For instance, Abs targeting histone H3 dimethylated on lysine 9 (H3K9me2) report conflicting signals in microscopy and genomics that either are exclusively found at the nuclear periphery [29–34] or also extend into the nuclear interior [35–40]. These conflicting results have fuelled debate about the degree to which H3K9me2 contributes to transcriptional repression [41, 42] or defines 3D chromatin structure [29, 31]. Similarly, Ab stainings for the nucleolar proteins fibrillarin and nucleophosmin produce microscopy signals that are either restricted to the surface of nucleoli or are also dispersed throughout its interior [43, 44]. Crucially, these discrepancies could be explained by certain Abs failing to penetrate into the interior of nuclear structures when applied to fixed or unfixed cells [44]. If so, this would indicate that a critical assumption behind many Ab-based chromatin mapping technologies—that Abs can physically reach all target epitopes in nuclei—could be fundamentally flawed.

Here, we systematically assess the ability of Abs to penetrate nuclei and sub-nuclear bodies, revealing a previously uncharacterized artifact, “Ab-trapping.” In Ab-trapping, Abs become trapped at the borders of structures with an abundant epitope. As a result, trapped Abs fail to diffuse inward, causing them to both miss internal epitopes and produce ectopic signals that mischaracterize protein distributions. Crucially, we demonstrate that this artifact broadly impacts many key chromatin targets—including widely used protein tags (e.g. GFP and HA), histone marks of heterochromatin (H3K9me2), and euchromatin (e.g. H4K8ac and H3K18ac), nucleolar proteins (e.g. B23 and Ki67), and transcription factors (PRRX1). Moreover, we show that Ab-trapping affects common assays, including immunofluorescence microscopy and CUT&Tag-seq, underscoring its widespread impact. Using computational simulations and experimental validation, we identify key parameters driving Ab-trapping and readily implementable protocol adjustments to mitigate it. Our work thus characterizes a crucial vulnerability in Ab-based assays that must be considered to accurately map epigenetic marks and chromatin proteins in the nucleus.

## Materials and methods

### Cell culture

Mouse TT2 embryonic stem cells (ESCs) and immortalized embryonic fibroblasts (iMEFs) were obtained from Yoichi Shinkai [45]. TT2 ESCs were cultured in Dulbecco’s modified Eagle’s medium (DMEM) supplemented with 10% fetal calf serum (FCS), 1× nonessential amino acids, sodium pyruvate, 2–mercaptoethanol, 1× penicillin/streptomycin, and 1000 U/ml leukemia inhibitory factor (LIF, Nacalai Tesque). iMEFs, 3KO (Setdb1, Suv39h1/2 triple knockout), and 5KO (Setdb1, Suv39h1/2, G9a, Glp quintuple knockout) iMEFs were maintained in the same medium as TT2 ESCs but without LIF.

The mouse myoblast cell line Pmi28 was grown in F-10 Nutrient Mixture (Ham) supplemented with 20% FCS and 1% penicillin/streptomycin. Naive J1 mESCs, mouse embryonic fibroblasts (MEFs), and H2A-HeLa cells were obtained from the Leonhardt laboratory. Wildtype HeLa cells for CUT&Tag-seq were obtained from Kalscheuer lab. Wildtype and H2B-eGFP-expressing HeLa cells were also obtained from Peter Cook lab [46]. Both MEFs and HeLa cells were cultured in DMEM supplemented with 10% FCS and 1% penicillin/streptomycin. J1 mESCs were cultured in serum-free media consisting of 50% N2B27, 50% DMEM/F12, 2i, 1000 U/ml recombinant LIF, 0.3% bovine serum albumin (BSA), 2 mM l-glutamine, and 100 U/ml penicillin. All cultures were maintained at 37°C and 5% CO₂ and regularly checked for mycoplasma contamination.

The COS-7 cells were cultured and transiently transfected with a plasmid encoding HA-tagged PRRX1 (S104G variant) under the control of a CMV promoter as described before [47].

### Animal husbandry

B6.Cg-Tg(Nrl-EGFP)1Asw/J (NRL-GFP) mice [48] were rederived by *in vitro* fertilization using cryopreserved sperm into C57Bl.6/J females. The line was maintained on a C57Bl.6/J background and used to isolate retinal rod cells. Other mouse samples were collected from the C57Bl.6/J strain sourced from Inotiv. All mice were housed in a centrally controlled environment with a 12-h light and 12-h dark cycle, temperature of 20°C–22.2°C, and humidity of 30%–50%. All mice had access to food and water *ad libitum*. Bedding, food, and water were routinely changed. All experiments involving animals were carried out following institutional guidelines as approved by LaGeSo Berlin and following the Directive 2010/63/EU of the European Parliament on the protection of animals used for scientific purposes.

### Tissue sampling

Fixation of tissues and preparation of cryosections was done as previously described [49]. Briefly, tissues were fixed with 4% formaldehyde in phosphate buffered saline (PBS) for 20–24 h, washed with PBS, incubated in sucrose with increasing concentrations (10%, 20%, and 30%), and transferred into embedding molds (Peel-A-Way Disposable Embedding Molds, Polysciences Inc., USA) filled with Jung freezing medium (Leica Microsystems). Tissue cryoblocks were frozen by immersing the molds into a −80°C ethanol bath and stored at −80°C. Cryosections of 16–20 μm thickness were cut using a Leica Cryostat (Leica Microsystems), collected on SuperFrost microscopic slides (SuperFrost Ultra Plus, Roth, Germany), immediately frozen, and stored at −80°C before use.

A retinal cell suspension for physical bisectioning was generated from four retinas of C57Bl.6/J adult mice. Retinas were dissociated using Papain Dissociation System (Worthington, #130-094-802) as described previously [50]. Briefly, four retinas from two mice were dissociated in papain solution for 60 min at 37°C while shaking at 700 rpm. Afterward, retinas were triturated with a 1-ml micropipette and transferred to a new tube containing a mix of EBSS, DNAse, and albumin-ovomucoid inhibitor. Cell suspensions were triturated again with a glass Pasteur pipette up to 10 times until tissue fragments were no longer visible. Finally, 250 µl of EBSS/albumin-ovomucoid inhibitor was added, and the single cell suspension was cleared of cell clumps by filtration (pluriStrainer Mini 70 μm). 1:1 ratio of 10% BSA/PBS was added to the final single cell suspension to form a cushion for centrifugation at 400 × *g* at 4°C for 10 min. Cells were then fixed in 4% paraformaldehyde in PBS (PFA) for 10 min at RT, after which the fixation was stopped with 130 mM glycine. Ten percent BSA was added in a 1:1 ratio and cells were pelleted by centrifugation (10 min, 400 × *g*, 4°C). Finally, cells were incubated in 30% sucrose/PBS solution at 4°C, scooped from tubes, and embedded in freezing medium as described for the tissues.

### CUT&Tag

#### Sample preparation

Samples were prepared for CUT&Tag assay as described previously with several modifications [20, 51]. HeLa cells were collected using TrypLE (Gibco, 12604021) and following centrifugation (5 min, 300 × *g*, RT), resuspended in CUT&Tag wash buffer [20 mM HEPES, pH 7.5 (Jena Bioscience, BU10675), 150 mM NaCl (Invitrogen, AM9760G), 0.5 mM spermidine (Sigma, S0266), 10 mM sodium butyrate (Sigma Aldrich, B5887), 1 mM PMSF (Thermo Fisher Scientific, 36978), Protease Inhibitor Cocktail (Roche, 04693132001)], and quantified using EVE automatic cell counter (NanoEntek).

For sampling rod cells, retinas from 2 month old NRL-GFP mice were dissociated using the Papain Dissociation System (Worthington, #130-094-802) as described above. Four retinas from two mice were used for one biological replicate. Fluorescence-Activated Cell Sorting (FACS) of single cells was performed on the BD FACS Aria II or Aria III Cell Sorter System with sample and plate cooling at 4°C. Forward-side scatter gating and GFP signal were used to sort rod perykaria into 15-ml falcons pre-coated with BSA and containing 5 ml of wash buffer for ~1 h. After sorting, rod cells were counted using a haemocytometer.

#### Tagmentation

CUT&Tag was performed as described previously with minor modifications [20, 51, 52]. Briefly, 11 μl Concanavalin A beads (BioMag, 86057) were equilibrated twice with 100 μl and then concentrated in 11 μl of binding buffer [20 mM HEPES, pH 7.5, 10 mM KCl (Invitrogen, AM9640G), 1mM CaCl_2_ (Merck, 102382), 1mM MnCl2 (Thermo Fisher Scientific, J63150.AD)]. One-hundred thousand cells were then bound to the beads by incubating for 10 min at RT with rotation. Beads were subsequently separated on a magnet and resuspended in 100 μl CUT&Tag Ab buffer [Wash buffer with 0.05% Digitonin (Milipore, 30410) and 2 mM ethylenediaminetetraacetic acid (EDTA; Invitrogen, 15575-038)]. Subsequently, 1 μl of primary Ab (Supplementary Table S1) or IgG control (Cell Signaling, 68860 for mouse; Cell Signaling, 2729 for rabbit; and Cell Signaling, 5415S for 48 h rod samples) was added and incubated on a rotator O/N (15 h) or 48 h at 4°C. After magnetic separation, beads were resuspended in 100 μl of Dig-wash buffer containing 1 μl of matching secondary Ab (Abcam, ab46540 for mouse; Antibodies-online, ABIN101961 for rabbit) and incubated for 1 h at 4°C with rotation. Following three washes with CUT&Tag Dig-wash buffer (wash buffer with 0.05% Digitonin), beads were resuspended in 100 μl CUT&Tag Dig-300 buffer (20 mM HEPES–KOH, pH 7.5, 300 mM NaCl, 0.5 mM spermidine, 0.01% digitonin, 10 mM sodium butyrate, 1 mM PMSF) with a 1:250 dilution of homemade 3xFLAG-pA-Tn5 preloaded with Mosaic-end adapters. After incubation at 4°C for 1 h with rotation, beads were washed four times with Dig-300 buffer and resuspended in 50 μl Tagmentation buffer [Dig-300 buffer 10 Mm MgCl_2_ (Invitrogen, AM9530G)]. Homemade spike-in-AmpR was added to the tagmentation buffer at a concentration of 33.6 fM [52]. Tagmentation was performed for 1 h at 37°C and subsequently stopped by adding 2.25 μl EDTA, 2.75 μl of 10% sodium dodecyl sulfate (SDS; Invitrogen, 15553-035), and 0.5 μl Proteinase K (Invitrogen, 25530049). DNA fragments were solubilized for 16 h at 55°C followed by 30 min at 70°C to inactivate residual Proteinase K. DNA fragments were finally purified and eluted with 25 μl of elution buffer (Zymo, D5205).

#### Library preparation and sequencing

NGS libraries were generated by polymerase chain reaction (PCR) amplifying the CUT&Tag DNA fragments with barcoded i5 and i7 primers [53] as described previously [51]. Following PCR, 1× volume of Ampure XP beads (Beckman Coulter) were mixed with the NGS libraries and incubated at RT for 10 min. After magnetic separation, beads were washed three times on the magnet with 80% ethanol, and the libraries were eluted with Tris–HCl, pH 8.0 (Roth, 9090.3). The quality of the purified NGS libraries was assessed with the D5000 tapestation (Agilent Technologies). The final libraries were sequenced on the Illumina NovaSeq X Plus platform with PE100 read length, yielding between 5 and 16 million fragments per sample.

#### NGS data processing

CUT&Tag sequencing was processed as previously described with several modifications [52, 54]. Briefly, sequencing adapters were trimmed using Trim Galore (v0.6.4) with the options --paired –nextera (https://github.com/FelixKrueger/TrimGalore). The trimmed reads were then aligned to the mouse genome (mm10) for rod cells or human genome (hg38) for HeLa cells using Bowtie2 (v2.3.5.1) [55] with --local --very-sensitive-local --no-mixed --no-discordant --phred33 -I 10 -X 2000. Aligned reads were filtered for mapped, paired-end reads (MAPQ ≥ 20) using samtools (v1.10) with view -f 2 -q 20 and then subsequently sorted. ENCODE-blacklisted regions [56] were removed by bedtools (v2.29.2) [57] with intersect -v. For visualization, normalized coverage tracks (bigWig) were generated with deepTools2 (v3.4.1) [58] using a bin size of 10 bp, read extension (-e), and CPM normalization, ignoring chrX and chrY. For merged tracks FASTQ files were concatenated from biological replicates. To visualize enrichment relative to the IgG control, log2 ratio tracks were generated using deepTools bigwigCompare (v3.4.1) [58]. Analogous 20-kb binned subtraction bedGraphs were then used for domain calling as previously described [59] with minor changes in parameters. Specifically, matrices were binarized with signal above a signal threshold considered positive and below as negative. Adjacent segments separated by gaps ≤200 kb were merged and, subsequently, regions <100 kb were removed.

To evaluate biological replicate consistency, consensus and unique domains were defined using bedtools intersect and subtract with -a parameter (v2.29.2) [57]. The consensus regions were used for all subsequent downstream analyses. To characterize signal distribution across distinct chromatin environments, the genome was segmented into four categories based on the presence or absence of ground-truth signals (LMNB1+/ab1220+, LMNB1+/ab1220−, LMNB1−/ab1220+, and LMNB1−/ab1220−) using bedtools intersect and subtract with -a parameter (v2.29.2) [57]. For distance analysis, Ab-defined domains were partitioned into 20 kb bins, and the linear distance to the nearest LMNB1 or ab1220 domain boundary was calculated using bedtools closest (v2.29.2) [57]. Importantly, the three anti-H3K9me2 antibodies (Abs) target the same epitope and therefore do not constitute statistically independent measurements, formal tests would yield inflated significance and were not applied. Differences between Abs are instead presented quantitatively, with effect sizes apparent from the plotted distributions.

Signal enrichment profiles across size-normalized LADs or ab1220 domains were generated using deepTools3 computeMatrix scale-regions and plotProfile (v3.5.1). Regions were scaled to 1 Mb with 1 Mb flanking sequences and a bin size of 10 kb. Finally, the correlation between standard (15 h) and extended (48 h) primary Ab incubation periods was assessed using deepTools3 multiBigwigSummary and plotCorrelation (with –skipZeros, –log1p, and –removeOutliers parameters) (v3.5.1). Pearson correlation coefficients were calculated for 2 kb bins using log-transformed raw bigwig files after excluding blacklisted regions.

Processed tracks and domain calling are available for visualization at https://genome-euro.ucsc.edu/s/Chudzik/AbTrapping (for isolated rod cells) and https://genome-euro.ucsc.edu/s/Chudzik/AbTrapping_hg (for HeLa cells).

### Immunostaining

#### Cultured and isolated cells

The primary and secondary Abs used in this study are presented in Supplementary Tables S1 and S2, respectively. Cells growing on coverslips were rinsed with PBS pre-warmed to 37°C and fixed with freshly made 4% formaldehyde, pH 7.0, for 10 min at RT. Cells were washed with PBS + 0.01% Tween20, 3× for 10 min at RT and permeabilized with 0.5% Triton X-100 in PBS for 10 min at RT. After washing with PBS for 10 min, coverslips were stored in PBS at + 4°C until use for up to 1 week. Primary and secondary Abs were diluted in blocking solution consisting of 4% BSA in PBS + 0.01% Tween20. Incubation with Abs was performed in dark chambers by placing coverslips up-side-down on drops of Ab solution loaded on Parafilm. If not otherwise indicated, incubations with primary and secondary Abs were for 1 h at RT (23°C–25°C). For DNA counterstain, DAPI or Hoechst 33342 was added to secondary Ab to final concentration of 2 or 1 µg/ml. Washings between and after Ab incubations were performed with PBS + 0.01% Tween20 pre-warmed to 37°C, 3× for 10 min each at 37°C. Coverslips were mounted on microscopic slides with antifade medium Vectorshield (VectorLabs), excess antifade was removed with soft tissue and coverslip edges were sealed with colorless nailpolish. For epitope reduction, gentle protein digestion with trypsin at 37°C for 10 min was applied with a concentration of 6.25 µg/ml (for H3K9me2) or 12.5 µg/ml (for B23).

The HA-PRRX1 fusion protein was detected with rabbit-anti-HA primary antibodies (Cell Signaling, #3724) diluted 1:100 as described before [47].

#### Immunofluorescence analogous to the CUT&Tag

One-hundred thousand single cells resuspended in 1 ml CUT&Tag wash buffer were attached to poly-lysine coated coverslips for 60 min at 4°C. Subsequently, non-attached cells were removed by two 5 min incubations at 4°C with the wash buffer. Incubation with Abs was performed in dark chambers by placing coverslips up-side-down on drops of Abs loaded on Parafilm. Primary Abs were used in the same quantities, buffers, and incubation times as described for CUT&Tag (see above). Donkey-anti-Rabbit Alexa555 (Invitrogen, A31572) and donkey anti-Mouse Alexa647 (Invitrogen, A-31571) were used as secondary Abs at 1:500 dilution. DAPI was added to the secondary Ab to a final concentration of 2 µg/ml. After both primary and secondary Ab incubations, coverslips were washed with a CUT&Tag Dig-Wash buffer at 4°C for 2× 10 min each. After final wash, cells were postfixed with 4% PFA, PBS + 0.01% Tween20, 2× for 10 min at RT, and mounted on microscopic slides, as described above for standard immunofluorescence.

#### Immunostaining of cryosections

Before staining, cryosections were dried out for 20 min at RT to fix sections on microscopic slides. For immunostaining of histone modifications, antigen retrieval was performed, which included re-hydration of sections in Na-citrate buffer for 5 min followed by heating up to 80°C in the same buffer for 15–30 min in a water bath. Sections were additionally permeabilized in PBS/0.5% Triton X-100 for 1 h. Primary and secondary Abs were diluted in blocking solution consisting of PBS, 2% BSA, 0.1% saponin, and 0.1% Triton X-100. Incubation with Abs was performed in dark wet containers under home-made glass chambers for 10–12 h at RT [60]. For nuclear counterstain, DAPI was added to secondary Ab to a final concentration of 2 µg/ml. Washings between and after Abs were performed with pre-warmed to 37°C PBS/0.01% Triton X-100, 3× for 30 min each at 37°C. Sections were mounted under coverslips with antifade and sealed as described above for cultured cells.

### Microscopy and image analysis

#### Confocal microscopy

Image stacks and single mid sections were acquired with either Leica or Nikon confocal microscopes. The TCS SP5 (Leica) confocal microscope was equipped with a Plan Apo 63/1.4 NA oil immersion objective and the Leica Application Suite Advanced Fluorescence Software (Leica). Z step size was adjusted to an axial chromatic shift and typically was either 200 nm or 300 nm. XY pixel size varied from 20 to 50 nm. Axial chromatic shift correction as well as building single gray-scale stacks, RGB-stacks, montages, and maximum intensity projections were performed using the ImageJ plugin StackGroom [61] available upon request. The Nikon confocal microscope A1 was equipped with Plan Apo lambda 100/1.45 NA oil immersion objective lens and the NIS-elements AR version 5.21.00 software (Nikon).

#### Time-lapse microscopy of immunostaining

MEFs grown on a 24-well glass-bottom plates (AGC Techno Glass) were fixed, permeabilized, and blocked with PBS containing 4% BSA and 0.01% Tween 20. Plates were then set on an inverted fluorescence microscope (Nikon, Ti-E) equipped with a 100x Plan Apo VC (NA 1.4) oil-immersion objective lens (Nikon), a spinning disc confocal unit (Yokogawa, CSU-W1), a laser unit (Chroma Technologies Japan, LDI-7 Laser Diode Illuminator), and an EM-CCD (iXon+; Andor; gain multiplier 300) operated by NIS-elements software ver. 5.11.03 (Nikon). Immediately after Ab mixture was added to a well, time-lapse recording at RT was started using 405-, 470-, and 555-nm laser lines with a 405/470/555/640NIR dichroic mirror, and 520/60, 600/50, and 690/50 emission filters. For double staining using CMA317 and AM39239, 100 μl of a mixture containing CMA317 (20 μg/ml), AM39239 (1:100 dilution), Alexa Fluor 488-conjugated goat anti-mouse Fcγ (Jackson ImmunoResearch; RRID, AB_2 338458, 20 μg/ml), Cy3-conjugated goat anti-rabbit IgG (H+L) (Jackson ImmunoResearch, RRID, AB_2337919; 20 μg/ml), and Hoechst 33342 (Nacalai Tesque; 04929-82, 4 μg/ml) was preincubated at room temperature for 2 h before adding to a well filled with 100 μl of PBS containing 4% BSA and 0.01% Tween 20. Fluorescence images were acquired every 5 min for 16.5 h.

For the comparison of mouse monoclonal Abs, CMA317 (IgG1 subclass) and ab1220 (IgG2a subclass) were distinguished using subclass-specific secondary Abs. A mixture containing both primary Abs (each 4 μg/ml) was preincubated with Cy3-conjugated anti-IgG1 (Jackson ImmunoResearch; goat anti-mouse IgG1, Fcγ subclass 1 specific; RRID, AB_2338461; 4 μg/ml) and Alexa Fluor 488-conjugated anti-IgG2a (Jackson ImmunoResearch; prepared by conjugating goat anti-mouse IgG1, Fcγ subclass 2a specific; RRID, AB_2338462; 4 μg/ml) for 30 min at room temperature prior to addition to the cells. Time-lapse imaging was performed every 5 min for 4 h. To quantify penetration speed, fluorescence intensity profiles were measured along a linear axis across the nucleus. Signal intensities were normalized to a range of 0 (minimum) to 1 (maximum) to allow for an ease of comparison of the penetration rates between the two mouse Abs.

To compare the penetration of regular Abs and nanobodies, HeLa cells expressing H2B-eGFP were incubated with a mixture of Alexa Fluor 568-labeled anti-GFP nanobody (Chromotech; GFP-Booster Alexa Fluor 568; 4 μg/ml) and rabbit anti-GFP Ab (Thermo Fisher Scientific; A-11122; 20 μg/ml) detected with Cy5-labeled goat anti-rabbit IgG (Jackson ImmunoResearch; RRID, AB_2337919; 40 μg/ml). Time-lapse imaging was initiated immediately upon Ab addition and was performed every 5 min for 12 h. Staining progression was monitored relative to the local H2B-EGFP expression levels to assess the impact of target density on probe diffusion and Ab-trapping.

#### FRAP

Fluorescence recovery after photobleaching (FRAP) was performed on HeLa cells expressing H2B-eGFP using a Nikon A1R confocal microscope with a 100x Plan Apo λ objective lens (NA 1.45) at room temperature, after overnight incubation with Alexa Fluor 568-labeled anti-GFP nanobody, rabbit anti-GFP IgG, and Cy5-labeled secondary Ab. After collecting three images (256 × 256 pixels; zoom x5; no averaging) using a low laser transmission (0.1% for 488-, 561-, and 640 nm) using a Nikon LU-N4 laser unit, a circular nuclear region was photobleached using 80% 561-nm and 30% 640-nm laser lines to target the Alexa Fluor 568 and Cy5 fluorophores, respectively. Images were further collected using the original settings for 30 min with 30-s intervals. Experiments were performed in the continued presence of the Abs in the surrounding medium. The relative intensities in a bleached area were measured using Image J, as described previously [46]. H2B-eGFP fluorescence was simultaneously recorded to monitor the distribution of the Ab target. Changes in Förster resonance energy transfer (FRET) between the EGFP donor and the Alexa Fluor 568 acceptor within the bleached area were observed before and after the bleaching.

#### Image quantification

**Figure 1.**
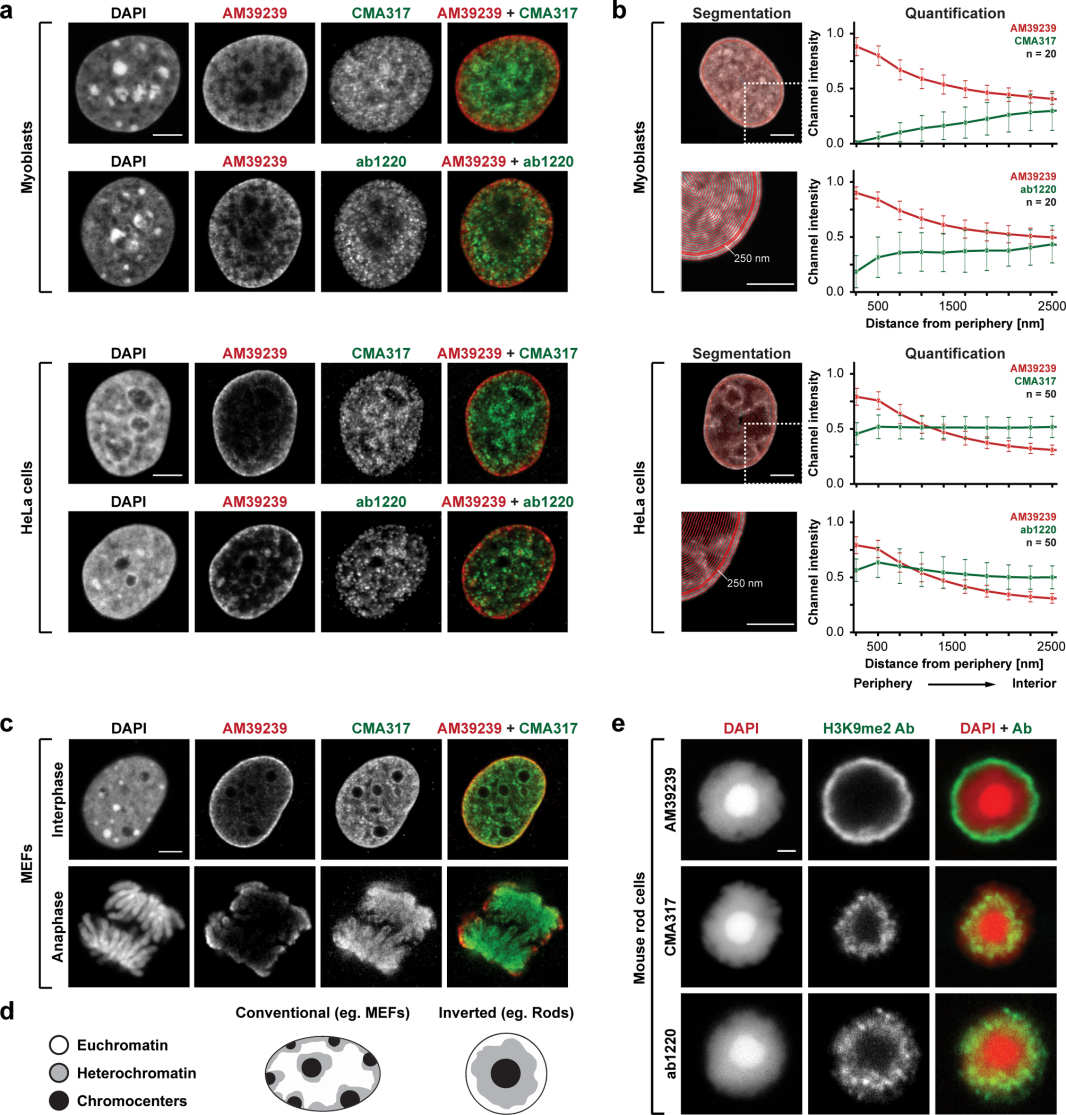
Anti-H3K9me2 Abs display contradicting immunofluorescence staining patterns. (**a**) Immunofluorescence staining with pairs of distinct anti-H3K9me2 Abs in cultured mouse (myoblasts) and human (HeLa) cell nuclei. (**b**) Quantification of immunofluorescence staining displayed in panel (a). Graphs show distribution of Ab signal intensities in concentric shells of AM39239 (red curves) and ab1220 or CMA317 (green curves). Nuclear segmentation into shells is depicted on the left with red contour lines. For details of nuclear segmentation and intensity normalization see the “Materials and methods” section. n, number of analyzed nuclei. (**c**) Immunofluorescence staining with pairs of anti-H3K9me2 Abs in interphase and mitotic cells in cultured MEFs. (**d**) Schematics of chromatin organization in conventional and inverted nuclei. (**e**) Immunostaining with single anti-H3K9me2 Abs in isolated rod perikarya. All images are confocal optical sections. Scale bars: panels (a)–(c), 5 µm and panel (e), 2 µm.

Images were processed using a custom Fiji macro to automate nuclear detection and spatial signal intensity analysis on the mid optical section. For experiments summarized in Figs 1a and b, 6b, and Supplementary Fig. S9a, to achieve better nuclear segmentation, two or three channels were summarized. Background subtraction, thresholding, and particle analysis were applied to detect nuclei and create regions of interest (ROIs). For each detected nucleus, concentric ring-shaped ROIs were generated by iteratively shrinking the initial ROI by 250 nm. Mean signal intensities were measured for all ring-shaped ROIs and visualized using Seaborn. The resulting plots display the mean intensity values for all measured samples per staining, normalized to the brightest signal in each channel. The *y*-axes represent the normalized mean signal intensity, error bars indicate the standard deviation across samples.

For experiments summarized in Fig. 7 and Supplementary Fig. S12 image stacks with several images (confocal sections) of nuclei per condition were pre-processed with a Gaussian filter prior to analysis. Nuclear intensity profiles were quantified in an automated procedure using a custom macro in FIJI (ImageJ 1.54p). For each image stack, nuclei were segmented using the “Li” auto-thresholding algorithm, and intensity profiles were extracted along the major axis of an ellipse fitted to the segmented mask. The profiles were cropped to exclude background regions (intensities below 10% of the intensity range) and normalized from 0 (min) to 1 (max). Within each condition, all profiles were interpolated to a uniform length, a mean profile was calculated, smoothed using a moving average filter (window size = 3), and plotted to visualize relative distribution changes. The number of analyzed nuclei typically varied between 20 and 50 per staining.

For quantification of HeLa cell nuclear areas (Supplementary Fig. S9b), mid sections of HeLa nuclei in S-phase were used after DAPI staining. Area measurements were performed in ImageJ using binary masks of mid sections.

### ELISA

The relative affinities of two mouse monoclonal Abs, CMA317 and ab1220, were measured using enzyme-linked immunosorbent assay (ELISA). Briefly, a 96-well plate was coated with 1 μg/ml synthetic H3K9me2 peptide [62] overnight and washed and blocked with PBS containing 0.1% Tween 20 and 1% BSA (PBST-BSA) for 1 h. After 3× washing with PBS containing 0.1% Tween 20, wells were incubated with a serial dilution series of Abs (one-third dilution series from 0.1 μg/ml) in duplicate in PBST–BSA for 1 h, cells were subsequently washed 3× in PBST, and incubated with peroxidase-conjugated AffiniPure™ goat anti-mouse IgG (1:10 000; Jackson ImmunoResearch; RRID, AB_10015289) for 1 h. After 3× washing with PBST, colorimetric assay was performed using 0.26 mg/ml O-phenylenediamine·2HCl (Fujifilm Wako Chemicals; 158-01671) in 0.1 M citrate buffer (pH 5.0) containing 0.01% hydrogen peroxidate (Fujifilm Wako Chemicals; 084-07441) by measuring absorbance at 450 nm using a plate reader (Thermo Fisher Scientific; Varioskan LUX).

To compare the binding affinity of AM39239 to CMA317, the concentration of the bound fraction to H3K9me2 was first estimated. This is because unpurified rabbit polyclonal Abs (AM39239) contain bulk IgG that do not bind to the target H3K9me2, unlike mouse monoclonal Abs. Briefly, Dynabeads™ MyOne™ Streptavidin T1 (Thermo Fisher Scientific; DB65601, 100 μl) were incubated with 1 μg of biotinylated histone H3K9 (Active Motif; 81043) or histone H3K9me2 peptide (Active Motif; 81046) for 1 h at room temperature. The beads were washed with PBST and mixed with rabbit polyclonal Ab AM39239 (12.5 μl beads per 1 μl Ab) for 2 h at room temperature. The supernatant was collected and the beads were washed three times with PBST and eluted using 1× SDS sample buffer without DTT [50 mM Tris–HCl (pH 6.8), 2% SDS, 10% glycerol, 0.01% bromophenol blue]. Input and supernatant samples (0.05 μl equivalent) and 10× concentrated bead eluate were separated on a 7.5% polyacrylamide gel (SuperSep™ Ace, 17 well pre-cast; Fujifilm Wako Chemicals; 191-14931) and stained with Bullet CBB Stain One (Nacalai Tesque; 21964-95). Relative band intensities were measured using ImageJ (https://imagej.net/ij/). The absence of H3K9me2-biding fraction from the supernatant was confirmed by immunofluorescence. IgG concentration in AM39239 was ∼5 mg/ml and the H3K9me2-bound fraction was ∼4% of total IgG (i.e. ∼0.2 mg/ml). The binding affinity of AM39239 to CMA317 was then compared under the saturated secondary Ab conditions by ELISA using a protocol modified in the following ways. The H3K9me2 peptide coating concentration was reduced to 0.3 μg/ml, and the Tween-20 concentration in the PBST–BSA was adjusted to 0.05%. The primary Ab incubation utilized a one-third serial dilution starting at 100 ng/ml for AM39239 and 10 μg/ml for CMA317. To detect these Abs, peroxidase-conjugated anti-rabbit IgG (for AM39239) (Jackson ImmunoResearch; RRID, AB_2307391) or anti-mouse IgG (for CMA317) (Jackson ImmunoResearch; RRID: AB_10015289) was used at a dilution of 1:250 or 1:500, respectively. All other reagents and detection steps remained identical to the procedure described above.

### Peptide arrays

The specificity of H3K9me2 Abs was assessed using the MODified™ Histone Peptide Array (Active Motif; 13005), according to the manufacturer’s instructions. After blocking the array slides with Blocking Buffer AM2 (Active Motif; 13006), they were incubated with 80 ng/ml AM39239 or 250 ng/ml CMA317 in 3 ml Blocking Buffer AM2 for 20 h. The slides were then washed three times with TBS containing 0.1% Tween-20 (TBST) (5 min each), incubated with 1:5000-diluted peroxidase-conjugated anti-rabbit IgG (Jackson ImmunoResearch; RRID, AB_2307391) or anti-mouse IgG (Jackson ImmunoResearch; RRID, AB_10015289) in 3 ml Blocking Buffer AM2 for 1 h, and washed again three times with TBST (5 min each). Chemiluminescence detection was performed using Western Lightning™ Plus-ECL (PerkinElmer; NEL104001EA).

### Computational modeling of antibody staining

We modeled Ab staining as a reaction-diffusion system of Abs (A) and antigens (B) inside the nucleus, which can be described by the following partial differential equations, where *c*_A_(*r, t*), *c*_B_(*r, t*), and *c*_AB_(*r, t*) are the spatiotemporal concentration fields of Ab, antigen, and Ab–antigen complex, respectively.


\begin{eqnarray*}
\frac{{\partial {{c}_{\mathrm{A}}}}}{{\partial t}} &=& {{D}_{\mathrm{A}}}{{\nabla }^2}{{c}_{\mathrm{A}}} - {{k}_{{\mathrm{on}}}}{{c}_{\mathrm{A}}}{{c}_{\mathrm{B}}} + {{k}_{{\mathrm{off}}}}{{c}_{{\mathrm{AB}}}},\\ \frac{{\partial {{c}_{\mathrm{B}}}}}{{\partial t}} &=& - {{k}_{{\mathrm{on}}}}{{c}_{\mathrm{A}}}{{c}_{\mathrm{B}}} + {{k}_{{\mathrm{off}}}}{{c}_{{\mathrm{AB}}}}, \\ \frac{{\partial {{c}_{{\mathrm{AB}}}}}}{{\partial t}} &=& {{k}_{{\mathrm{on}}}}{{c}_{\mathrm{A}}}{{c}_{\mathrm{B}}} - {{k}_{{\mathrm{off}}}}{{c}_{{\mathrm{AB}}}}.
\end{eqnarray*}

*D*_A_ is the diffusion constant of Abs. The diffusion coefficients of the antigen (*D*_B_) and the Ab–antigen (*D*_AB_) complex were set to be zero, as the antigen considered is on histones that are bound to cross-linked chromatin. *k*_on_ and *k*_off_ are rate constants for the Ab binding and unbinding. For initial conditions, antigen was uniformly distributed in a sphere of radius *R*_0 _= 5 µm at concentration c^0^_B_; Abs were distributed outside of this sphere in a concentric shell of *R*_1 _= 75 µm at concentration c^0^_A_. To mitigate the effect of stiffness for numerically solving the coupled equations, we smoothed the initial concentrations *c*_A_ (*r, t* = 0), *c*_B_(*r, t* = 0) by a hyperbolic tangent function and scaling factor ε was set to be 0.2:


\begin{eqnarray*}
{c}_{\mathrm{A}} (r,t = 0) &=& \frac{c_{\mathrm{A}}^0}{2}\left[1 + {\mathrm{tanh}} \left(\frac{{r}^{2} - R_0^2}{\varepsilon} \right) \right],\\{c}_{\mathrm{B}} (r,t = 0) &=& \frac{c_{\mathrm{B}}^0}{2}\left[1 - {\mathrm{tanh}} \left(\frac{{r}^{2} - R_0^2}{\varepsilon} \right)\right].
\end{eqnarray*}

We discretized the problem in spherical coordinates, assuming spherical symmetry of the nucleus, which reduces the three-dimensional system to a one-dimensional problem, using a spatial grid spacing of 0.01 μm. To address the challenges of solving stiff equations when *K*_d_ (i.e. *k*_off_/*k*_on_) is small, we utilized the Julia DifferentialEquations package, using the Rodas5P solver. This solver is a fifth-order A-stable Rosenbrock method, specifically designed for stiff problems, and is complemented by a stiff-aware fourth-order interpolant for improved accuracy and stability [63, 64].

## Results

### Anti-H3K9me2 Abs produce conflicting staining patterns in standard immunofluorescence

We sought to clarify whether H3K9me2 is found exclusively at the nuclear periphery [29–34] or also operates in the nuclear interior [35–40]. We therefore systematically mapped the distribution of H3K9me2 using immunofluorescence microscopy and three different anti-H3K9me2 Abs. These included two commercial Abs, Active Motif AM39239 and Abcam ab1220, and the CMA317 Ab generated in H. Kimura’s laboratory [65].

We first co-stained mouse myoblasts and human HeLa cells with rabbit AM39239 mixed with either mouse CMA317 or mouse ab1220. Both mouse Abs produced staining that was consistently distributed throughout nucleoplasm, including at the nuclear periphery, the periphery of nucleoli, and other loci (Fig. 1a). However, AM39239 was strikingly confined to the nuclear periphery with only a residual gradient of staining toward the nuclear interior. Quantifying the radial signal distribution confirmed these contradictory staining results, validating that the signal of AM39239 is more peripherally enriched than the signal from the other two anti-H3K9me2 Abs (Fig. 1b). These distinct staining patterns of the three anti-H3K9me2 Abs also proved highly consistent. AM39239 staining was persistently more restricted to the periphery than CMA317 or ab1220, both when each Ab was used separately (Supplementary Fig. S1a) and when applied across multiple cultured cell-types (Supplementary Fig. S1b). Moreover, we find similar differences at different stages of the cell cycle. While CMA317 or ab1220 stained through condensed mitotic chromosomes during anaphase, AM39239’s signal was almost entirely restricted to their periphery (Fig. 1c). The peripheral enrichment of AM39239 was also consistently observed between several tested Ab lots (Supplementary Fig. S1c).

These contradictory Ab behaviors are also mirrored in isolated rod cell photoreceptors, a unique cell-type with a radically inverted nuclear organization where heterochromatin occupies the nuclear interior (Fig. 1d) [6, 66]. Here, CMA317 and ab1220 stained the layer of heterochromatin surrounding the central chromocenter in the nuclear interior. In contrast, AM39239 specifically marked the peripheral euchromatin layer, where no H3K9me2 staining is expected ((Fig. 1e). Critically, these contradictory results match conflicting H3K9me2 stainings previously reported for these same Abs in rods, with CMA317 and ab1220 Abs marking the nuclear interior [38] and AM39239 the nuclear periphery [31].

Thus, different Abs reproducibly report highly distinct distributions of H3K9me2 in multiple contexts from conventional and inverted nuclei to mitotic chromosomes.

### Peripherally restricted H3K9me2 staining is a technical artifact

We next sought to determine which staining pattern of the three anti-H3K9me2 Abs is artifactual and what causes them to behave differently. We noted three aspects of AM39239’s staining that were inconsistent with previous observations. First, the restriction of AM39239’s staining to a narrow rim of peripheral chromatin in conventional nuclei was unexpected given the reported enrichment of H3K9me2 at nucleolar-associated domains in the nuclear interior [67]. Second, the observation of H3K9me2 enrichment in the euchromatic periphery of inverted rod nuclei contradicted its established role in transcriptional repression [42]. Third, nearly exclusive peripheral H3K9me2 staining in anaphase chromosomes seemed unlikely considering that euchromatin and heterochromatin are not spatially segregated in mitotic chromosomes [68]. Combined, we reasoned that AM39239’s tendency toward peripheral accumulation is an artifact, while CMA317 and ab1220 Ab staining report H3K9me2’s true distribution.

We first hypothesized that AM39239 displays unspecific binding to off-target epitopes, thereby causing incorrect staining. To test this, we examined Ab specificity using modified histone peptide arrays. These confirmed that AM39239 and CMA317 bind H3K9me2, though the binding of both Abs was sensitive to neighboring H3S10ph and H3T11ph (Supplementary Fig. S2). However, while CMA317 showed no off-target binding, AM39239 displayed cross-reactivity with some non-H3K9me2 peptides that contain H3K9me3 or H3K27me2 (Supplementary Fig. S2).

We therefore tested the localization of off-target AM39239 binding by immunofluorescence staining of MEFs that lack the five H3K9 methyltransferases (5KO-MEFs) [45]. Both mouse Abs CMA317 and ab1220 displayed no signal in 5KO-MEFs, demonstrating that they are highly specific to H3K9me2. In contrast, rabbit AM39239 exhibited a residual non-specific signal throughout entire nuclear chromatin (Supplementary Fig. S3a). As H3K9me3 is also depleted in these cells, this background likely corresponds to the H3K27me2 cross-reactivity detected on the peptide array. Crucially, however, AM39239’s signal enrichment at the nuclear rim was eliminated in the 5KO cells, indicating that the peripheral signal is dependent on the H3K9me2 epitope. Supporting this, prolonged BSA blocking, a standard method to minimize non-specific protein interactions [69], did not alter the peripheral enrichment of AM39239 (Supplementary Fig. S3b). The distinct staining patterns of the three anti-H3K9me2 Abs are thus not due to differences in epitope specificity.

We instead postulated that artifactual AM39239 staining arises from the differing abilities of anti-H3K9me2 Abs to penetrate the nucleus. To examine this, we attempted to physically “break” the nuclear surface to improve the accessibility of Abs to chromatin. We generated a suspension of briefly fixed retinal cells, embedded them in a cryoblock, and prepared 14-µm thick cryosections for staining with AM39239. Cell nuclei lying entirely within the sections maintained their integrity and exhibited characteristic AM39239 nuclear rim staining. In contrast, nuclei that were physically bisected by the sectioning, as visible by disruptions in the DAPI channel, showed staining of internal chromatin that was now exposed (Supplementary Fig. S3c). This indicates that the AM39239 Ab can successfully stain heterochromatin throughout the nucleus, but only when it has direct access to the nuclear interior. This finding raised the question of what could prevent AM39239 from accessing epitopes inside the nucleoplasm.

We thus last investigated whether the peripheral staining by AM39239 is linked to differences in its binding affinity to H3K9me2, compared to ab1220 and CMA317. Comparing the two mouse Abs by ELISA revealed that ab1220 exhibits ∼27-fold lower affinity than CMA317 (Supplementary Fig. S4a). Subsequently, to compare the rabbit AM39239 with mouse CMA317, we utilized a modified ELISA with saturated secondary Abs to normalize detection across species. This revealed that AM39239 displays a ∼34-fold higher binding affinity to H3K9me2 than CMA317 (Supplementary Fig. S4b–e). Based on the previously determined binding coefficient of CMA317 (*K*_d_ = 1.5 × 10^−8^ M) [65], we estimated that *K*_d_ of ab1220 is ∼4 × 10^−7^ M and AM39239 is ∼4.4 × 10^−10^ M. Thus, AM39239, which displays the strongest peripheral accumulation, also has the highest binding affinity of the three anti-H3K9me2 Abs.

Collectively, this suggests that the persistent peripheral staining with AM39239 is due to insufficient Ab penetration into the nucleus, which could be related to its ∼34- to 918-fold higher binding affinity to the H3K9me2 epitope.

### Computer simulations identify parameters affecting Ab penetration

**Figure 2.**
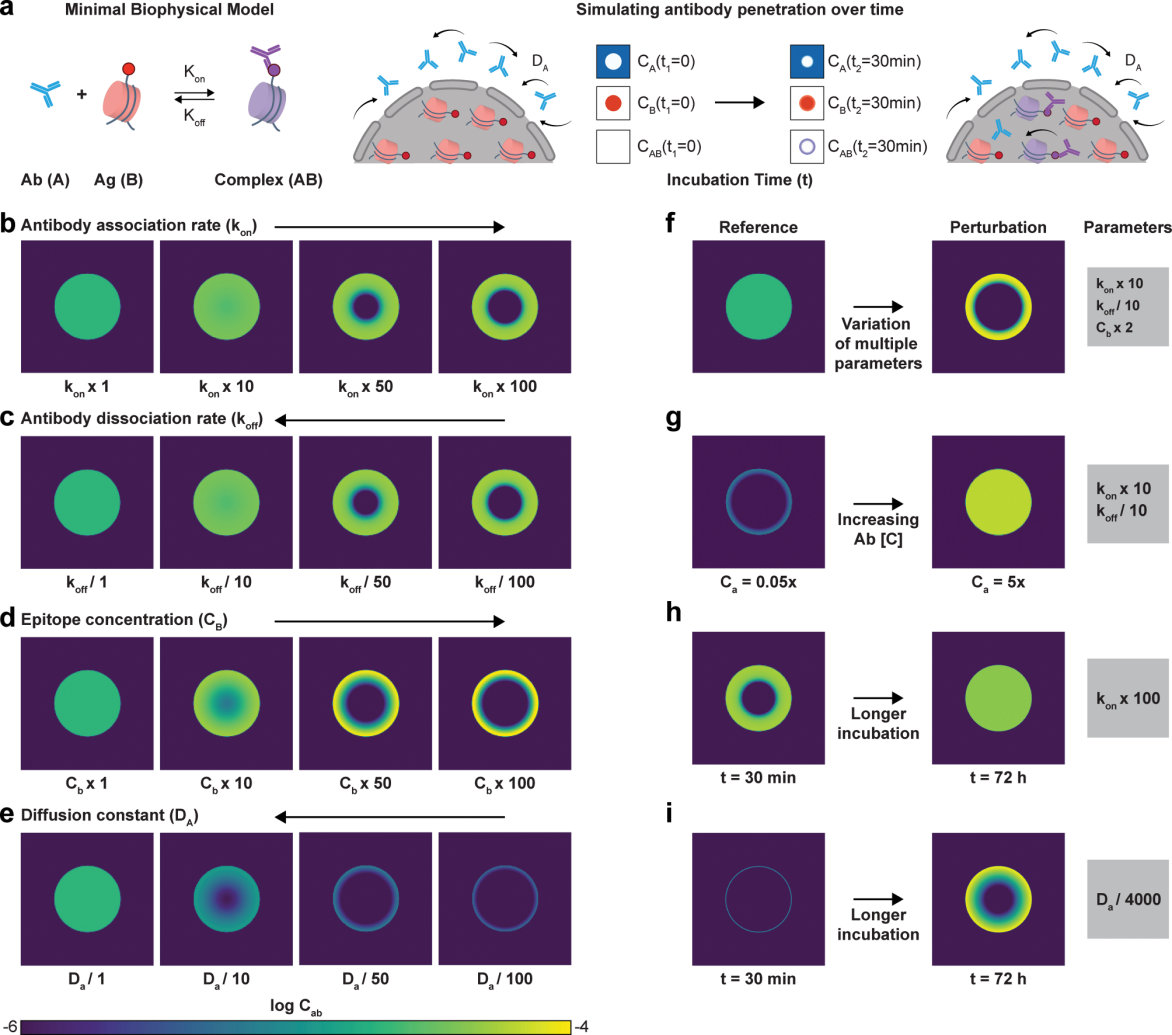
Computational modeling recapitulates peripherally restricted Ab staining. (**a**) Minimal biophysical model. Left: Ab (A) and Ag (B) bind and unbind reversibly with rate *k*_on_ and *k*_off_. Right: at time 0, Abs are primarily located outside the nucleus, and they diffuse into the nucleus with a diffusion constant *D*_A_. The model describes the spatial positioning of freely diffusing Ab (*C*_A_), antigen (*C*_B_), and antigen bound to Ab (*C*_AB_) as three concentration fields, each illustrated at time *t* = 0 and after a 30-min incubation. *C*_AB_ at time *t* = 30 min in simulations where parameters for *k*_on_ (**b**), *k*_off_ (**c**), *C*_B_ (**d**), and *D*_A_ (**e**) are individually altered to produce Ab-trapping (See Supplementary Fig. S5). (**f**) Trapping at time *t* = 30 min can also be realized by smaller alterations that affect combinations of *k*_on_, *k*_off_, and *C*_B_. (**g**) A 100-fold increase of Ab concentration [C] results in uniform signal distribution under conditions that otherwise results in peripheral signal. (**h**) A 100-fold increase of *k*_on_ leads to strong trapping at 30 min, which can be abolished with a prolonged incubation time *t* = 72 h. (**i**) A 4000-fold decrease in the diffusion constant results in strong trapping at time *t* = 30 min with the gradient remaining pronounced even at time *t* = 72 h.

We sought to decipher how Ab affinity is connected to artifactual peripheral staining by building a minimal computational model of Ab binding dynamics in a simulated nucleus. We modeled staining of the nucleus over time as a reaction-diffusion process, similar to models used to optimize tissue-scale staining [70, 71]. Specifically, we approximated the nucleus as a 10 µm diameter sphere with an antigen (Ag) uniformly distributed throughout its volume. We assumed that an Ab moves according to a specific diffusion coefficient (*D*_A_). We also assumed Ab–Ag interactions are governed by their association (*k*_on_) and dissociation (*k*_off_) rates (Fig. 2a). The baseline values for these parameters were adapted from published datasets. These included (i) the known affinity of the CMA317 Ab, (ii) the estimated H3K9me2 epitope concentration per single mammalian nucleus, and (iii) the diffusion coefficient for IgG molecules in aqueous solution (Supplementary Fig. S5a and see the “Materials and methods” section) [65, 72–74]. In these conditions, our simulations show Abs progressively penetrate the nucleus over time to ultimately produce a uniform distribution of signal within 30 min (Supplementary Fig. S5b and c).

We then varied one parameter at a time to determine how they individually affect Ab penetration after 30 min. Increasing Ab *k*_on_ by 100-fold from the baseline or decreasing Ab *k*_off_ by 100-fold abolished internal penetration (Fig. 2b and c). This indicates that proper Ab penetration is blocked when epitope binding affinity is high, matching our observations for the higher affinity of AM39239. Similarly, Ab penetrance into the nuclear interior could also be inhibited by increasing the Ag concentration (*C*_b_) by 50-fold or decreasing the diffusion coefficient (*D*_A_) by 50-fold (Fig. 2d and e). Failed Ab penetration also arises when smaller changes are made to several variables simultaneously. Internal staining was also prevented by simultaneously reducing Ab dissociation (*k*_off_) 10-fold while increasing Ab binding (*k*_on_) 10-fold and Ag concentration (*C*_b_) 2-fold (Fig. 2f). Thus, a peripheral staining artifact can arise by individually or synergistically modulating parameters that impact Ab penetration.

We finally investigated parameters that could later be experimentally adjusted to potentially resolve failed Ab penetration, namely Ab concentration and incubation time. Increasing Ab concentration by 100-fold produced a uniform staining pattern under simulation conditions that previously produced peripheral staining (Fig. 2g), while decreasing Ab concentration exacerbated it (Supplementary Fig. S5d and e). Likewise, extending the simulated incubation time from 30 min to 72 h similarly reversed peripheral staining (Fig. 2h). Artifactual peripheral staining is thus predicted to be mitigated, in some cases, by increasing Ab concentration or incubation time. However, we note that modifications, like extended 72 h incubation times, cannot restore Ab penetration if the various parameters that cause it are too extreme (Fig. 2i).

Taken together, our simulations argue that Ab performance in a closed volume is strongly influenced by multiple parameters, including epitope abundance, *k*_on_, and *k*_off_ that define Ab affinity, and diffusion rate. Combinations of low affinity, low epitope concentration, and high diffusion consistently improve nuclear interior staining. In contrast, combinations of high affinity, high epitope concentration, and a low diffusion constant result in peripherally restricted staining of simulated nuclei. However, this failed penetration can be reversed by increasing Ab concentration and incubation time. We termed this phenomenon Ab-trapping and next sought to experimentally test these computational predictions.

### High epitope abundance contributes to Ab-trapping

**Figure 3.**
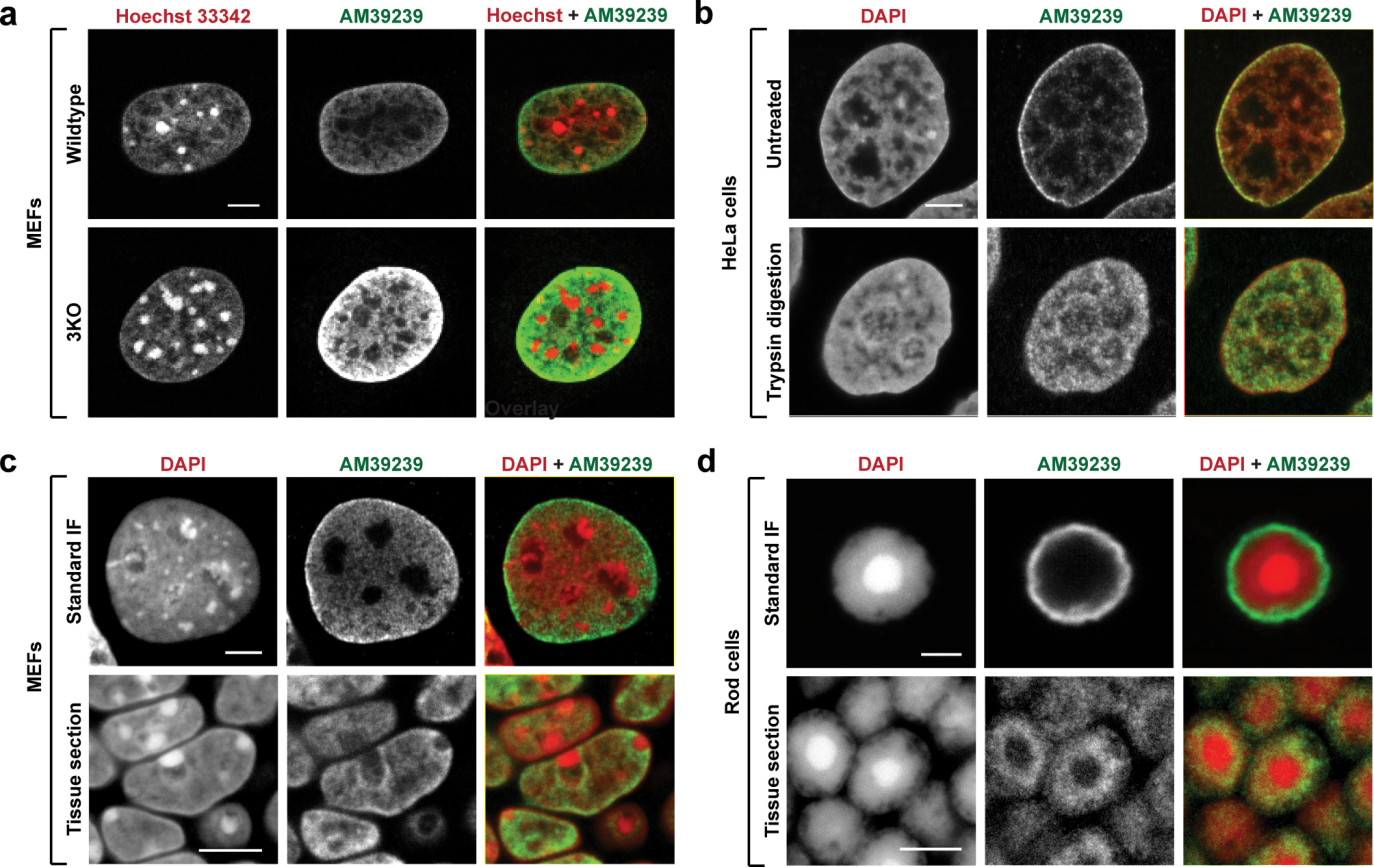
High epitope abundance contributes to Ab-trapping of AM39239. (**a**) Immunofluorescence staining with AM39239 in WT and 3KO MEFs. Ab signal in the nuclear interior is substantially increased in 3KO cells, although the gradient from the periphery toward the center is still present. (**b**) Immunofluorescence staining with AM39239 in HeLa nuclei without (top) and with (bottom) nonspecific reduction of proteins by trypsin digestion that notably enhances internal staining. (**c, d**) Immunofluorescence staining with AM39239 in single cells (top layers) and in tissue cryosections (bottom layers). In cultured MEFs [panel (c), top] and isolated rod photoreceptors [panel (d), top], the nuclear staining is peripheral. In sections of embryonic limb [panel (c), bottom], in addition to the peripheral rim, the Ab stains the nuclear interior including the nucleolar periphery. In sections of retina [panel (d), bottom], the Ab stains the internal heterochromatin layer. All images are single optical sections. Scale bars: 5 μm.

We began by testing whether high epitope concentration blocks Ab penetration. We did so by decreasing the abundance of H3K9me2 in three ways prior to detection with AM39239. First, we applied immunofluorescence to 3KO-MEFs, which lack three of the five H3K9 methyltransferases—Suv39H1, Suv39H2, and SetDB1—resulting in a 30% reduction of H3K9me2 levels [45]. As predicted, in the 3KO cells, the peripheral staining with AM39239 was substantially but not completely abrogated, and staining became more intense and uniformly distributed throughout the nucleoplasm (Fig. 3a). Second, we applied a gentle trypsin digestion, which should remove histone tails like those marked with H3K9me2. This limited digestion similarly promoted a more uniform labeling of chromatin in the nuclear interior (Fig. 3b). Finally, we performed immunofluorescence staining in tissue sections. Here, prolonged sample crosslinking and antigen retrieval by heating (see the “Materials and methods” section) are expected to greatly reduce epitope abundance relative to standard immunofluorescence with the short crosslinking used for cultured cells. Accordingly, performing antigen retrieval prior to immunostaining with AM39239 restores nucleoplasmic signal in cryosections of embryonic limb (Fig. 3c) and adult retinas (Fig. 3d and Supplementary Fig. S6). Together, this supports that high epitope abundance is a major contributor to Ab-trapping, at least for the anti-H3K9me2 Ab AM39239.

### High Ab affinity contributes to Ab-trapping

We further tested the prediction that Ab-trapping is linked to AM39239’s high affinity for H3K9me2, which is 34- and 918-fold greater than that of CMA317 and ab1220, respectively (Supplementary Fig. S4). To do so, we examined the penetration dynamics of these two Abs by following progressive Ab staining in fixed cells over time using time-lapse microscopy. The Abs were preincubated with fluorescently labeled secondary Abs and mixed with Hoechst 33342, before incubating with fixed permeabilized cells. Strikingly, peripheral nuclear signal was initially observed for both Abs and even for Hoechst staining. Full nuclear staining with CMA317 was then achieved after several hours, whereas staining with AM39239 was not completed even after 12 h (Supplementary Fig. S7 and Supplementary Video S1). We observed a similar trend when comparing CMA317 with the lower-affinity ab1220 using subclass-specific secondary Abs (Supplementary Fig. S8a). We found that ab1220 stained the nuclear interior more rapidly than CMA317 (Supplementary Fig. S8b and Supplementary Video S2). However, ab1220 and CMA317 eventually fully penetrated the nucleus after 12 h, displaying highly overlapping staining patterns (Supplementary Fig. S8c). Collectively, these results confirm that higher affinity promotes Ab-trapping by impeding the diffusion of Abs from the periphery to the interior.

### Ab-trapping impacts genomic assays

We next set out to determine how Ab-trapping affects genomics assays that rely on Ab diffusion in nuclei, focusing on CUT&Tag [20]. CUT&Tag differs substantially from standard immunofluorescence, as Abs are applied to unfixed or only lightly fixed nuclei in distinct permeabilization and Ab incubation buffers. We therefore first tested whether the three anti-H3K9me2 Abs behave similarly under these alternative conditions. To do so, we performed immunostaining under CUT&Tag conditions in isolated rod photoreceptors, where H3K9me2 is confined to the nuclear interior. As under standard immunofluorescence, ab1220 correctly labeled internal heterochromatin, while AM39239 remained restricted to the nuclear periphery, consistent with Ab-trapping (Supplementary Fig. S9a). Strikingly, CMA317 now also produced an exclusively artifactual peripheral signal (Supplementary Fig. S9a), despite not being subjected to Ab-trapping under standard immunofluorescence conditions (Fig. 1e).

To investigate the basis for this variability, we compared nuclear morphology in unfixed and fixed cells incubated in PBS or CUT&Tag buffers. Nuclei exposed to CUT&Tag buffers were markedly smaller than those in PBS, especially without fixation, indicating reduced nuclear volume (Supplementary Fig. S9b). This compaction would be expected to increase the local concentration of H3K9me2 epitopes, which our computational modeling and experimental perturbations identify as a key driver of Ab-trapping (Fig. 2d). Together, these findings suggest that CUT&Tag conditions are particularly permissive for Ab-trapping. More broadly, they highlight that susceptibility to Ab-trapping can vary substantially for the same Ab depending on the experimental conditions in which it is applied.

**Figure 4.**
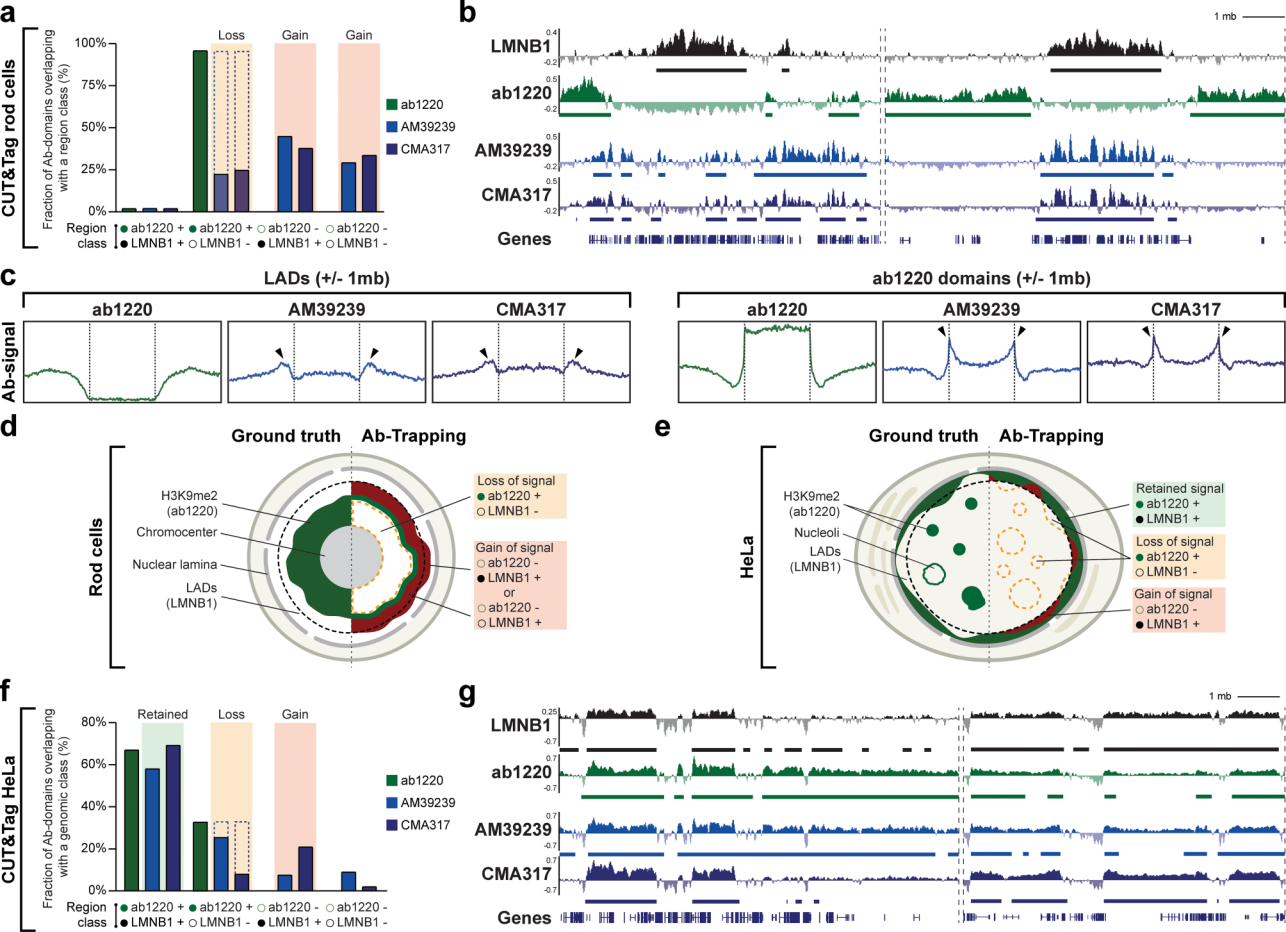
Ab-trapping mischaracterizes H3K9me2 genomic profiles in a cell-type-specific manner. (**a**) Quantification of the genomic distribution of Ab-defined domains across region classes categorized by the presence or absence of LMNB1 or ab1220 signal. Dashed outlines above the AM39239 and CMA317 bars indicate the ab1220 reference level, highlighting the fraction of missing signal relative to the ground truth. (**b**) CUT&Tag tracks in rod cells for the indicated Abs. Tracks represent the average of two biological replicates (log2 signal after IgG control subtraction) with ref-seq genes shown below. Domain calling for each sample is indicated below the corresponding track. (**c**) Metaplots showing the distribution of signal for the indicated Abs in rod cells across size-normalized LADs (left) or ab1220 domains (right). One megabase of flanking regions are shown in both directions. Arrowheads mark enrichment of AM39239 and CMA317 signal at domain boundaries. (d, e) Schematic illustrating the impact of Ab-trapping on signal distribution in inverted rod cell nuclei (**d**) and conventionally organized HeLa cells (**e**). (**f, g**) Matching quantification and genome browser tracks of CUT&Tag-seq as in panels (a) and (b), but in HeLa cells.

We further examined how Ab-trapping by CMA317 and AM39239 manifests at the molecular level in CUT&Tag-seq. We performed CUT&Tag in mouse rod photoreceptors using the three anti-H3K9me2 Abs together with an anti-LMNB1 Ab to map peripheral LADs. In all cases, domains called for each Ab were highly concordant between two biological replicates (Supplementary Fig. S9c). As expected of inverted rod nuclei, 96% of ab1220 domains were located outside of LADs (ab1220+, LMNB1−), consistent with H3K9me2 near exclusively occupying the nuclear interior (Fig. 4a). In contrast, the trapped Abs CMA317 and AM39239 captured only a subset of this internally positioned H3K9me2, overlapping with only a fraction of ab1220+ LMNB1− regions. Thus, Ab-trapping prevents efficient recovery of internal H3K9me2 signal in CUT&Tag, matching our microscopy results.

However, CUT&Tag also revealed a second and less intuitive consequence of Ab-trapping. Both trapped Abs also produced unexpected ectopic gains of signal in regions where H3K9me2 is normally absent, including at those in the nuclear interior (ab1220−, LMNB1−) and nuclear periphery (ab1220−, LMNB1+) (Fig. 4a). Inspection of genome browser tracks suggested that this ectopic enrichment arises at the boundaries between H3K9me2-positive heterochromatin domains and euchromatic LADs (Fig. 4b). We therefore plotted Ab signals across LADs and ab1220-defined H3K9me2 domains. CMA317 and AM39239 signals peaked just beyond LAD borders and at the edges of H3K9me2 domains (Fig. 4c). These patterns are consistent with Abs becoming trapped at the first abundant epitopes they encounter, thereby generating ectopic CUT&Tag signal to inappropriately extend into neighbouring loci (Fig. 4d). In the case of H3K9me2 in rods, this position lies where internal heterochromatin meets peripheral euchromatin.

To test this, we finally examined whether both the loss and gain of signal Ab-trapping artifacts also appear in conventionally organized nuclei by repeating H3K9me2 and LADs CUT&Tag-seq in HeLa cells (Fig. 4e). As expected for these cells, 67% of H3K9me2 domains overlapped with LADs (ab1220+, LMNB1+), while 33% were found in the nuclear interior (ab1220+, LMNB1−) (Fig. 4f and g). However, consistent with Ab-trapping, CMA317 recovered less internal H3K9me2 (ab1220+, LMNB1−) and displayed ectopic signal in LADs with weak or absent H3K9me2 (ab1220−, LMNB1+ and ab1220−, LMNB1−). AM39239 displayed a similar tendency but to a lesser extent. Thus, in both inverted and conventional nuclei, Ab-trapping reduces internal signal while producing ectopic signal where the first abundant epitopes are encountered, which depends on the cell’s nuclear organization.

In summary, this demonstrates that Ab-trapping mischaracterizes the genomic distributions of chromatin features in CUT&Tag-seq, both through loss and ectopic gain in signal.

### Ab-trapping affects multiple Abs, epitopes, and structures

Our modeling suggests that Ab-trapping can in principle affect any Ab or cellular structure with epitopes that are confined to a limited volume. We thus examined several alternative scenarios where artifactual peripheral staining could emerge in a manner dependent on Ab affinity and/or epitope abundance.

**Figure 5.**
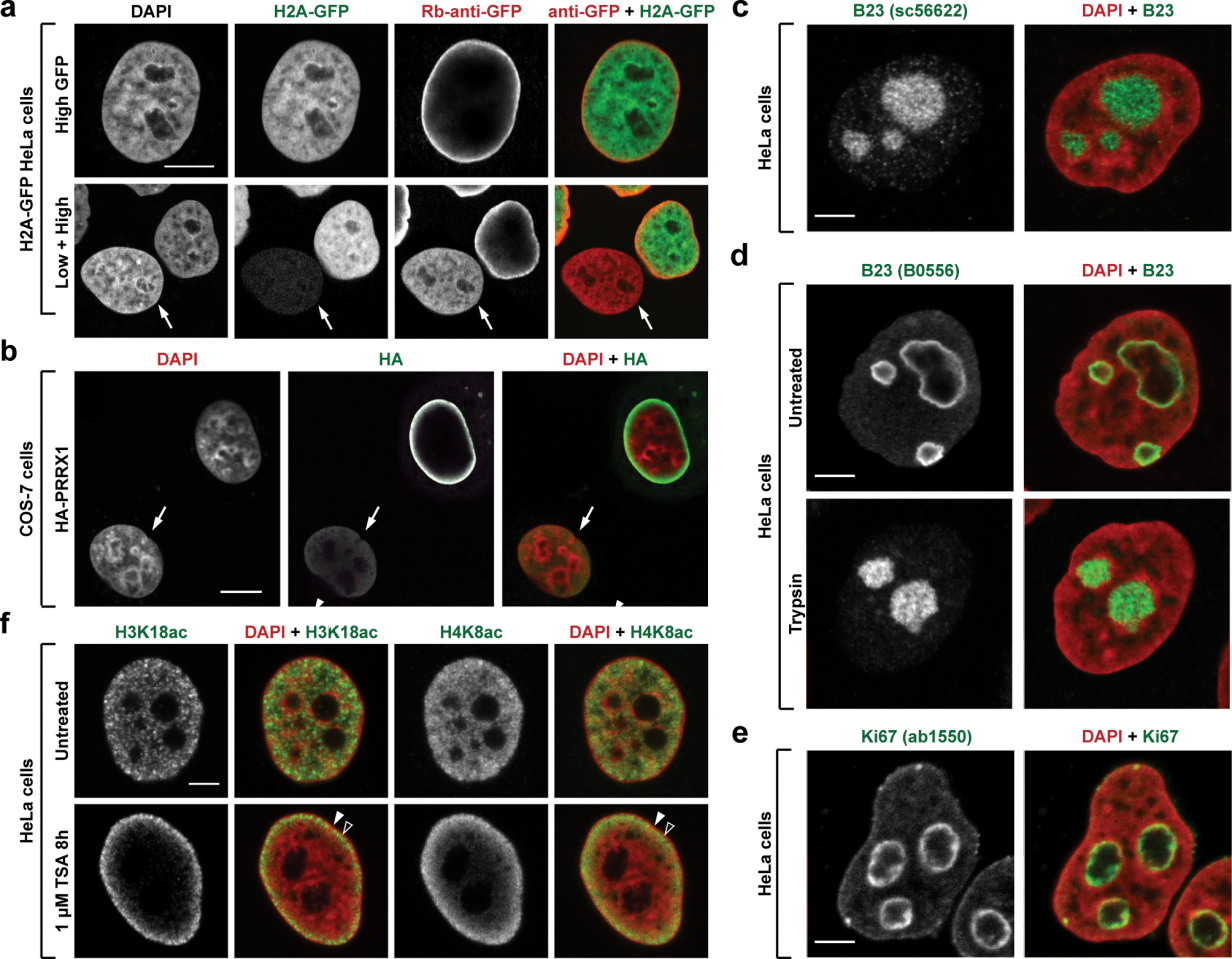
Multiple Abs and target epitopes exhibit the trapping effect. (**a**) Immunostaining of HeLa cell nuclei with variable expression of H2A-GFP with anti-GFP Ab. Peripheral Ab-trapping is observed in highly expressing H2A-GFP cells but not in cells with lower expression (arrow). (**b**) Immunostaining of COS-7 cell nuclei expressing HA-tagged transcription factor PRRX1. Note the false nuclear peripheral staining in highly expressing cell and nucleoplasm staining in cell with lower expression (arrow). Immunostaining of HeLa cells with anti-B23 (**c, d**) and anti-Ki67 (**e**) Abs. Note that the false B23 immunostaining [panel (d), top] can be avoided by using a different Ab (sc-56622) [panel (c)] or mild protein digestion [panel (d), bottom]. (**f**) Immunostaining with anti-H3K18ac or anti-H4K8ac Abs in HeLa cells without (top) and with increased histone acetylation via Trichostatin A treatment (bottom). Homogeneous staining with H3K18ac and H4K8ac Abs throughout the internal euchromatin becomes peripheral after the increase in epitope abundance. Note that in contrast to AM39239 (Fig. 1) or anti-GFP (Fig. 5a) Ab-trapping at the extreme nuclear periphery, euchromatin Ab-trapping (empty arrowheads) occurs more internally than the peripheral rim of heterochromatin (solid arrowheads). All images are single optical sections. Scale bars: panels (a) and (b) 10 µm, and panels (c) and (d) 5 µm.

We initially applied standard immunofluorescence to a HeLa cell line that heterogeneously expresses H2A-GFP at variable levels across the cell population. Strikingly, the anti-GFP Ab A-11122 artifactually stained the nuclear periphery in cells with high H2A-GFP expression. By contrast, lower expressing cells with a reduced GFP epitope abundance were unaffected (Fig. 5a). In parallel, HeLa cells were co-stained with an anti-GFP nanobody that binds GFP with a single monomeric variable domain and is thus 10-fold smaller than rabbit IgGs [75]. The nanobody stained GFP throughout the entire nucleoplasm independently of GFP expression level (Supplementary Fig. S10a and b). Time-lapse imaging confirmed that the IgG stains nuclear interior significantly slower than the nanobody, whether applied separately or in combination (Supplementary Fig. S10c). However, FRAP revealed minimal fluorescence recovery for both probes, indicating no substantial difference in their dissociation (*k*_off_) rates (Supplementary Fig. S10d), suggesting that the nanobody bypasses Ab-trapping due to its greater diffusion coefficient (*D*_A_). Thus, even anti-GFP Abs are subject to Ab-trapping under the synergistic effects of high epitope abundance, high Ab affinity, and slow diffusion.

We further examined other tagged proteins for Ab-trapping. Staining the HA-tagged transcription factor PRRX1 results in a nucleoplasm-wide signal when its expression is low but only in false peripheral staining when its expression is high (Fig. 5b). Likewise, a previous study demonstrated that anti-GFP or anti-FLAG Abs display Ab-trapping at the nucleolar surface when fibrillarin-GFP and EGFP-FLAG-B23 are overexpressed in HeLa cells [43, 44]. Matching this, we found that the abundant endogenous B23 protein also displays Ab-trapping depending on the Ab used. Specifically, the sc-56622 Ab stains nucleoli uniformly, whereas the alternative Sigma B0556 Ab stains only the nucleolar periphery causing a typical Ab-trapping effect that is abrogated after mild digestion (Fig. 5c and d). Similarly, staining of another abundant intra-nucleolar protein Ki67 with Abcam ab15580 was also restricted to the nucleolar periphery (Fig. 5e). Thus, Ab-trapping can affect multiple tagged and endogenous proteins in distinct cellular structures.

We last tested for potential Ab-trapping in other abundant histone modifications that instead define euchromatin. Under normal conditions, Abs against H4K8ac or H3K18ac displayed enriched signals in the nuclear interior in HeLa cells. However, elevating the concentration of acetylated histones in HeLa cells following treatment with the HDAC inhibitor Trichostatin A increased labeling to an Ab-trapping-like sub-peripheral rim at the border of euchromatin (Fig. 5f).

Ab-trapping can therefore affect Abs targeting a wide range of epitopes, including both endogenous and ectopically expressed proteins within the nucleus and nucleolus.

### Factors that mitigate Ab-trapping

**Figure 6.**
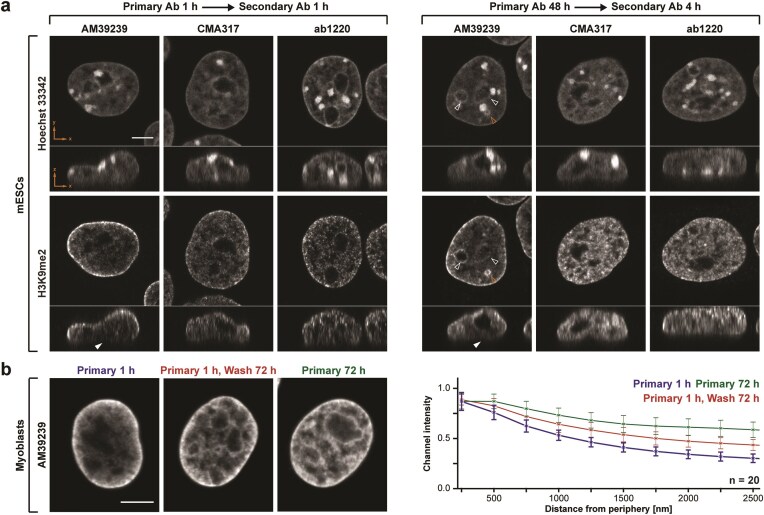
Increasing incubation time with and between Abs mitigates Ab-trapping. (**a**) Immunostaining with three anti-H3K9me2 Abs with standard (left) and extended (right) incubation times. AM39239 stains the nuclear periphery closest to the coverslip/slide only in the extended incubation (solid white arrowheads in XZ sections). Note AM39239 staining at the nucleolar periphery (empty white arrowheads) and nuclear channels formed by the invaginated nuclear envelope (empty gold arrowheads) in extended incubations. (**b**) Immunostaining with AM39239 (left) and quantification of radial signal distributions (right) in three conditions: (i) 1 h primary Ab incubation followed by 30 min wash, (ii) 1 h primary Ab followed by 72 h wash, and (iii) 72 h primary Ab incubation followed by 30 min wash. All images are single optical sections. Scale bars: 5 µm.

Our computational modeling (Fig. 2h) and time-lapse imaging (Supplementary Figs S7 and S8) suggested that increasing the time available for Ab diffusion could mitigate Ab-trapping. We therefore tested the impacts of prolonged Ab incubation and washing times. In immunofluorescence, both longer primary Ab incubation (48 or 72 h) and extended washing (72 h) improved AM39239 penetration, though some partial peripheral trapping remained (Fig. 6). However, in CUT&Tag-seq, extending AM39239 or CMA317 incubation times to 15 or 48 h did not alleviate the artifact, again revealing the sensitivity of Ab-trapping to experimental conditions (Supplementary Fig. S11).

**Figure 7.**
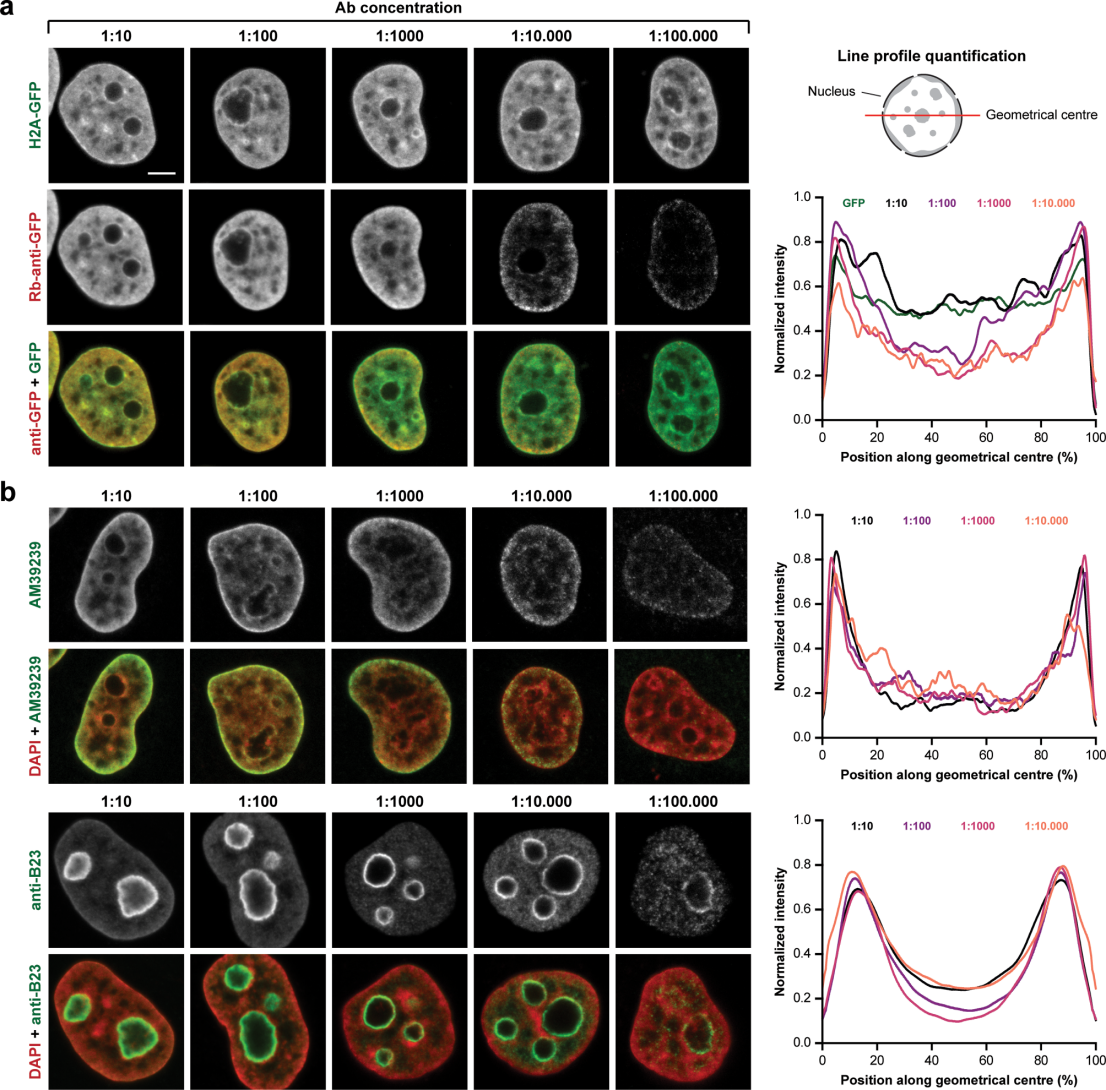
Increasing primary Ab concentration can mitigate trapping for some Abs. Immunostaining of H2A-GFP in highly expressing cells (**a**), H3K9me2 with AM39239 (**b**, top), and nucleophosmin (B23) (**b**, bottom) in nuclei of HeLa cells at indicated dilutions. Representative single optical sections (left) and corresponding line profile quantifications (right) are shown. The 1:100 000 condition is omitted from quantification graphs due to the low signal-to-noise ratio. Schematic of the line profile analysis over the nuclear geometrical center is depicted above the graphs. All images are single optical sections. Scale bars: 5 µm.

We also tested the other computational prediction that increasing Ab concentration can overcome trapping by saturating peripheral epitopes. This intervention proved context-dependent. Increasing anti-GFP Ab concentration progressively reduced peripheral trapping in H2A-GFP HeLa cells, ultimately recapitulating underlying GFP signal at the highest concentration (Fig. 7a). In contrast, high concentrations of AM39239 or anti-B23 failed to eliminate peripheral trapping, presumably due to the high abundance of endogenous epitopes relative to Abs present (Fig. 7b). Varying secondary Ab concentration also had no effect, indicating that the primary Ab remains the limiting factor (Supplementary Fig. S12).

Thus, prolonged Ab incubation, extended washing, or increased Ab concentration can mitigate Ab-trapping in some cases. However, the success of these interventions depends on the specific Ab, target epitope abundance, and experimental context.

## Discussion

The biomedical sciences struggle with a “reproducibility crisis,” which has been in part attributed to the unreliable performance of Ab-based assays [76–78]. As a result, major efforts have been undertaken in the last decade to systematically identify and mitigate these issues. Here, we characterize an undescribed immunolabeling artifact, Ab-trapping, that profoundly mischaracterizes how chromatin features are distributed in the genome and nuclei. Importantly, we show that this artifact is not caused by a lack of Ab-specificity or other previously reported problems in Ab-based assays. Instead, Ab-trapping is driven by failed Ab diffusion caused by the interplay between (i) a high Ab affinity and (ii) a high concentration of an epitope that is confined to a limited volume.

We show that Ab-trapping represents a significant flaw in likely any assay relying on Ab penetration into whole nuclei, including immunofluorescence microscopy and CUT&Tag-seq. Given its widespread impact, it is essential to broadly raise awareness of this artifact across research disciplines. Indeed, microscopists, who have long applied Ab to whole nuclei, have anecdotally recognized peripheral staining patterns as artifacts in some instances [43, 44] (personal communications of Evgenya Popova, Sergei Grigoryev, Robert Schneider, and others). However, this is not always the case [31, 34, 79]. In contrast, the genomics community has only relatively recently adopted technologies relying on Ab penetration into whole unfixed cells or isolated nuclei (e.g. CUT&RUN/ChIC, CUT&Tag, pA-DamID, and MAbID) [18–22]. Genomics users are thus potentially less prepared to identify Ab-trapping, which is crucial considering these technologies underlie most state-of-the-art single-cell and spatial epigenome mapping methods [20, 22–25]. Ultimately, recognizing and addressing Ab-trapping will significantly enhance the accuracy and reproducibility of experiments across microscopy and genomics alike.

In demonstrating Ab-trapping, our study helps to resolve existing conflicts in the published literature. In the absence of Ab-trapping, we show that H3K9me2 marks heterochromatin regardless of whether it is positioned at the nuclear periphery or not. In cells with conventional genome organization, H3K9me2 is enriched at the periphery but also labels a significant fraction of chromatin in the nuclear interior, as was reported previously [35–40]. Conversely, in rod cells with inverted nuclear organization, H3K9me2 relocates to the nuclear interior together with the heterochromatin it is physically associated with. Combined, this demonstrates that H3K9me2 is primarily a mark of repressive heterochromatin rather than spatial position in the nucleus as some studies have concluded [29, 31]. This example of a prominent histone modification highlights how Ab-trapping can lead to incorrect conclusions concerning genome biology. Moreover, we demonstrate that Ab-trapping can affect diverse chromatin targets from transcription factors and histone modifications to nucleolar proteins and protein tags. Thus, Ab-trapping has significant potential to misinform the study of broad areas of chromatin biology and so cannot be ignored.

While Ab-trapping has been recognized by the microscopy community in some cases, a model of artifactual peripheral staining at the cellular scale has been lacking until now. With this model, we demonstrate that Ab-trapping emerges from a small set of physical properties, such as binding kinetics, diffusion rates, and epitope density, without necessitating more complex biological explanations. By isolating these variables, the model establishes a mechanistic rationale for the artifact and provides a clear set of testable parameters that we were able to validate experimentally. It also paves the way to future more elaborative models that expand on the simplifying assumptions made here. First, cell nuclei are not perfectly spherical, so artifacts arising from the staining process could be influenced by a target geometry. Second, we do not model fluid advection or non-specific binding events, both of which could affect staining dynamics. Third, considerable uncertainty remains regarding reference parameters, including Ab affinity and diffusion coefficients. Finally, we did not model bivalent or cooperative binding. Incorporating such details may enable computational screening to avoid Ab-trapping and other artifacts across a broad range of staining scenarios.

It is important to emphasize that susceptibility to Ab-trapping is not an intrinsic property of an Ab, but rather a context-dependent phenomenon influenced by specific experimental parameters. Our results demonstrate that while some Abs, such as ab1220, perform consistently across applications, others, such as CMA317 and AM39239, are highly sensitive to the specific experimental environment. Thus, our findings argue against relying on prior benchmarks, e.g. Ab specificity and standard immunofluorescence that are universal “sanity checks.” Instead, they highlight the necessity of validating Ab performance under the exact biochemical and physical conditions of the intended assay.

We would finally like to alert the scientific community that multiple commercially available Abs against nuclear proteins display apparent Ab-trapping evident even from representable images on the manufacturer’s websites. These are exemplified by Abs from Thermo Fisher Scientific for H1 and H4 acetylation, active motif for histone H3, and Abcam for H3K27ac (Supplementary Table S3). Importantly, this does not mean that these Abs lack specificity. Rather, their high affinity and/or elevated abundance of their target epitopes likely predisposes them to Ab-trapping. Consequently, such Abs can still produce reliable results if appropriate measures are employed to mitigate Ab-trapping. To assist researchers in recognizing and mitigating Ab-trapping, we recommend the following straightforward best practices:

1. **Validation of genomics assays by microscopy**. For any bulk or single cell genomic method relying on Ab diffusion (e.g. CUT&Tag), perform a parallel immunofluorescence control, and apply recommendations listed below to determine whether this signal is a result of Ab–trapping.
2. **Peripheral staining caution**. Immunostaining restricted to the periphery of the nucleus, nucleolus, or other structures with a confined volume should be interpreted with caution. If possible, use a different Ab (or tagged protein expression) to confirm the peripheral localization.
3. **Expression level variability**. When immunodetecting ectopically expressed proteins via protein tags, ensure that a sufficient number of cells covering a wide range of expression levels are analyzed. If a peripheral signal is observed in only a fraction of highly expressing cells, it is likely the result of Ab-trapping caused by high epitope abundance.
4. **Optimization of staining conditions**. When using an untested primary Ab, test various parameters, such as different staining and washing times (from 1 to 72 h). Likewise, various fixation and incubation conditions might influence Ab-trapping, e.g. differential performance of CMA317 in unfixed cells and CUT&Tag buffer versus fixed cells and PBS buffer.
5. **Confirmation by using cryosections**. Whenever possible, validate staining patterns observed in isolated or cultured cells by using tissue cryosections where longer crosslinking and antigen retrieval reduces epitope abundance.
6. **When a trapping Ab is the only one available**. To mitigate the Ab-trapping effect, we recommend increasing times for primary Ab incubation or washing between primary and secondary Abs. Alternatively, for an Ag reduction, a mild digestion with a protease can greatly improve staining results.

## Supplementary Material

gkag615_Supplemental_Files

## Data Availability

All data reported in this paper are available upon request. Sequencing data generated in this study are available at the NCBI Gene Expression Omnibus, GEO: GSE293376. Processed CUT&Tag tracks are also available at https://genome-euro.ucsc.edu/s/Chudzik/AbTrapping (for isolated rod cells) and https://genome-euro.ucsc.edu/s/Chudzik/AbTrapping_hg (HeLa cells) for visual exploration in UCSC. Code for computational modeling has been deposited at https://github.com/Fudenberg-Research-Group/Ab-trapping and Zenodo at https://doi.org/10.5281/zenodo.20072870.

## Notes

Present address: Faculty of Agriculture, Shizuoka University, Shizuoka, 422-8529, Japan

Present address: Division of Cell and Molecular Biology, The Institute of Cancer Research, London, SW3 6JB, United Kingdom
