# Supplementary material for: Antibody-trapping presents a widespread pitfall for microscopy and genomics in the nucleus
Source: Nucleic Acids Res. 2026 Jun 27;54(12):gkag615. doi: 10.1093/nar/gkag615 (PMC13309790; doi:10.1093/nar/gkag615)
Supplement: gkag615_Supplemental_Files [file gkag615_supplemental_files.zip › Chudzik_Supplementary_Data_Proofs.pdf]

Supplemental Figures and Supplemental Figure Legends

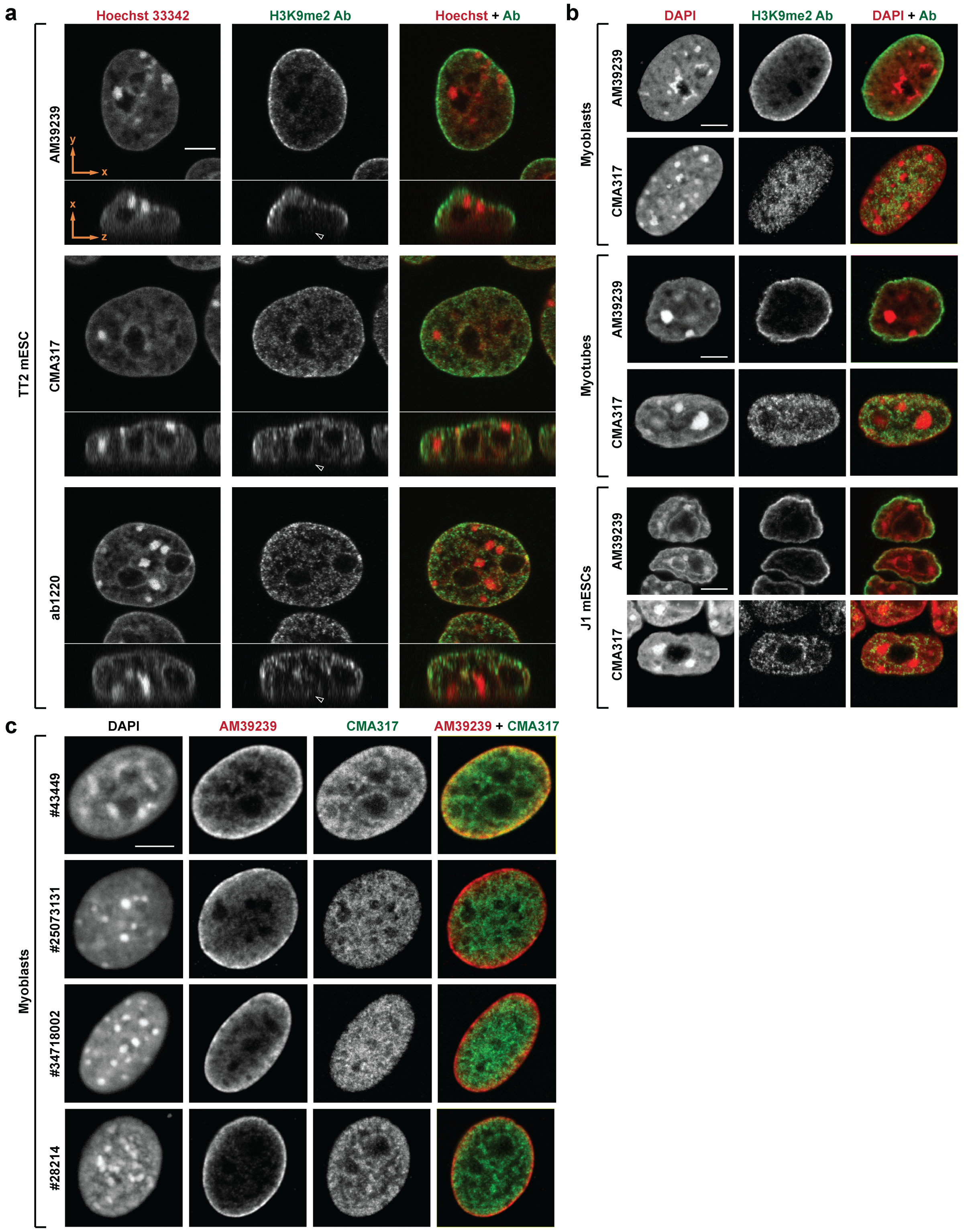

Supplementary Figure S1. Immunofluorescence using three different anti-H3K9me2 Abs in multiple cell-types. a, Staining with single anti-H3K9me2 Abs in TT2 mESCs nuclei. XY (top) and XZ (bottom) views are shown. Arrowheads in XZ sections point at the nuclear periphery adjacent to the substrate, which lacks staining with AM39239. **b**, Dual staining with anti-H3K9me2 Abs, AM39239 and CMA317, in nuclei of cultured mouse myoblasts, myotubes (differentiated from myoblasts) and J1 mESCs. **c**, Immunofluorescence staining with different lots of AM39239 (indicated on the left) in combination with CMA317 in nuclei of mouse myoblasts. All images are single optical sections. Scale bars: 5 μm.

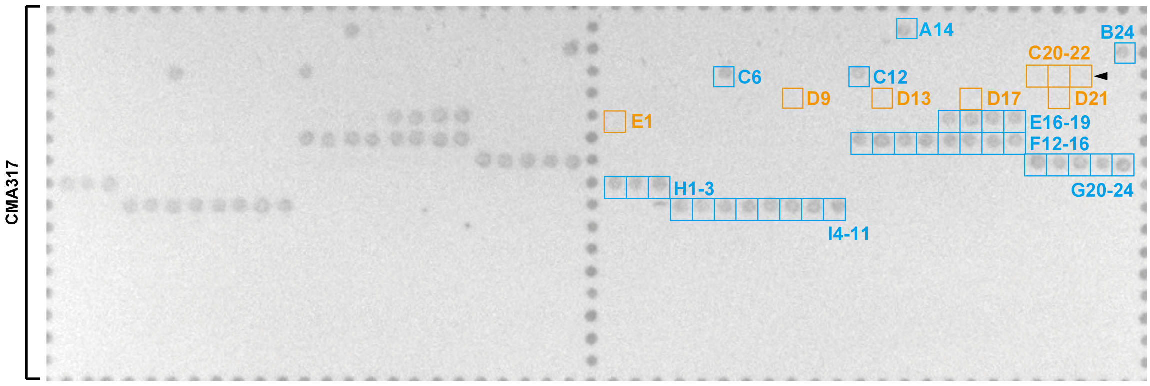

A14 H3 1-19 K9me2
B24 H3 1-19 R8me2s, K9me2
C6 H3 1-19 R8me2a, K9me2
C12 H3 1-19 R8citr, K9me2
C20 H3 1-19 K9me2, S10P
C21 H3 1-19 K9me2, T11P
► C22 H3 1-19 K9me2, K14ac
D9 H3 1-19 R8me2s, K9me2, S10P
D13 H3 1-19 R8me2s, K9me2, T11P
D17 H3 1-19 R8me2a, K9me2, S10P
D21 H3 1-19 R8me2a, K9me2, T11P
E1 H3 1-19 R8me2a, K9me2, S10P, T11P
E16 H3 1-19 R2me2a, K4me1, K9me2
E17 H3 1-19 R2me2a, K4me2, K9me2
E18 H3 1-19 R2me2a, K4me3, K9me2
E19 H3 1-19 R2me2a, K4ac, K9me2
F12 H3 1-19 K4me1, R8me2s, K9me2
F13 H3 1-19 K4me2, R8me2s, K9me2
F14 H3 1-19 K4me3, R8me2s, K9me2
F15 H3 1-19 K4ac, R8me2s, K9me2
F16 H3 1-19 K4me1, R8me2a, K9me2
G20 H3 1-19 R2me2s, K4me1, R8me2s, K9me2
G21 H3 1-19 R2me2s, K4me2, R8me2s, K9me2
G22 H3 1-19 R2me2s, K4me3, R8me2s, K9me2
G23 H3 1-19 R2me2s, K4ac, R8me2s, K9me2
G24 H3 1-19 R2me2a, K4me1, R8me2s, K9me2
H1 H3 1-19 R2me2a, K4me2, R8me2s, K9me2
H2 H3 1-19 R2me2a, K4me3, R8me2s, K9me2
H3 H3 1-19 R2me2a, K4ac, R8me2s, K9me2
I4 H3 1-19 R2me2s, K4me1, R8me2a, K9me2
I5 H3 1-19 R2me2s, K4me2, R8me2a, K9me2
I6 H3 1-19 R2me2s, K4me3, R8me2a, K9me2
I7 H3 1-19 R2me2s, K4ac, R8me2a, K9me2
I8 H3 1-19 R2me2a, K4me1, R8me2a, K9me2
I9 H3 1-19 R2me2a, K4me2, R8me2a, K9me2
I10 H3 1-19 R2me2a, K4me3, R8me2a, K9me2
I11 H3 1-19 R2me2a, K4ac, R8me2a, K9me2

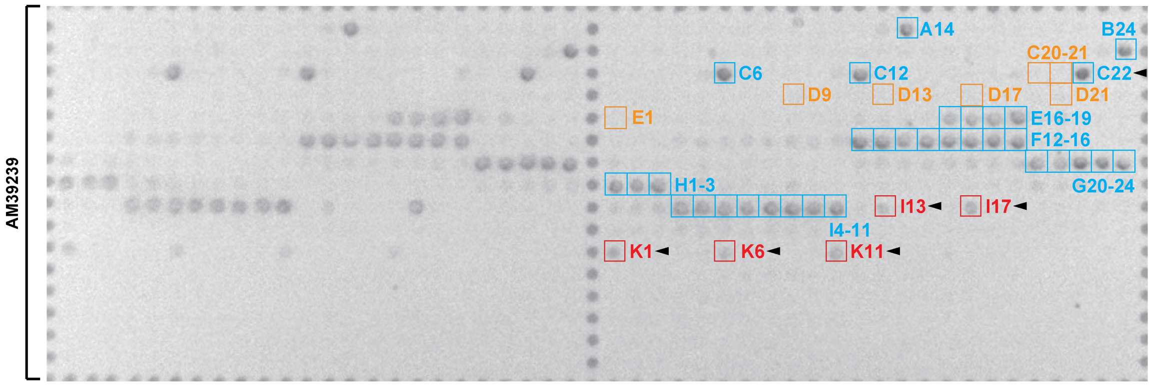

A14 H3 1-19 K9me2
B24 H3 1-19 R8me2s, K9me2
C6 H3 1-19 R8me2a, K9me2
C12 H3 1-19 R8citr, K9me2
C20 H3 1-19 K9me2, S10P
C21 H3 1-19 K9me2, T11P
► C22 H3 1-19 K9me2, K14ac
D9 H3 1-19 R8me2s, K9me2, S10P
D13 H3 1-19 R8me2s, K9me2, T11P
D17 H3 1-19 R8me2a, K9me2, S10P
D21 H3 1-19 R8me2a, K9me2, T11P
E1 H3 1-19 R8me2a, K9me2, S10P, T11P
E16 H3 1-19 R2me2a, K4me1, K9me2
E17 H3 1-19 R2me2a, K4me2, K9me2
E18 H3 1-19 R2me2a, K4me3, K9me2
E19 H3 1-19 R2me2a, K4ac, K9me2
F12 H3 1-19 K4me1, R8me2s, K9me2
F13 H3 1-19 K4me2, R8me2s, K9me2
F14 H3 1-19 K4me3, R8me2s, K9me2
F15 H3 1-19 K4ac, R8me2s, K9me2
F16 H3 1-19 K4me1, R8me2a, K9me2
G20 H3 1-19 R2me2s, K4me1, R8me2s, K9me2
G21 H3 1-19 R2me2s, K4me2, R8me2s, K9me2
G22 H3 1-19 R2me2s, K4me3, R8me2s, K9me2
G23 H3 1-19 R2me2s, K4ac, R8me2s, K9me2
G24 H3 1-19 R2me2a, K4me1, R8me2s, K9me2
H1 H3 1-19 R2me2a, K4me2, R8me2s, K9me2
H2 H3 1-19 R2me2a, K4me3, R8me2s, K9me2
H3 H3 1-19 R2me2a, K4ac, R8me2s, K9me2
I4 H3 1-19 R2me2s, K4me1, R8me2a, K9me2
I5 H3 1-19 R2me2s, K4me2, R8me2a, K9me2
I6 H3 1-19 R2me2s, K4me3, R8me2a, K9me2
I7 H3 1-19 R2me2s, K4ac, R8me2a, K9me2
I8 H3 1-19 R2me2a, K4me1, R8me2a, K9me2
I9 H3 1-19 R2me2a, K4me2, R8me2a, K9me2
I10 H3 1-19 R2me2a, K4me3, R8me2a, K9me2
I11 H3 1-19 R2me2a, K4ac, R8me2a, K9me2
► I13 H3 1-19 R2me2s, K4me2, R8me2a, K9me3
► I17 H3 1-19 R2me2a, K4me2, R8me2a, K9me3
► K1 H3 1-19 K27me2
► K6 H3 1-19 R26me2s, K27me2
► K11 H3 1-19 R26me2a, K27me2

Supplementary Figure S2. Evaluation of anti-H3K9me2 Abs specificity by peptide array. Evaluation of CMA317 (top) and AM39239 (bottom) specificity using MODified™ Histone Peptide Arrays (Active Motif). Each array contains two identical halves (left and right), with the full set of modified histone peptides spotted in each half. The details of modified peptides are listed below the array images, including H3K9me2 peptides that were detected by antibodies (blue), those that were not detected (orange), and peptides without H3K9me2 that were detected (red). Arrowheads indicate peptides showing differential binding between AM39239 and CMA317. Both Abs bind to the majority of peptides containing H3K9me2 (blue boxes). Neither Ab bound to H3K9me2 peptides that also harbor H3S10ph or H3T11ph (orange boxes). AM39239 displayed cross-reactivity with some peptides lacking H3K9me2, including those harboring H3K9me3 or H3K27me2 in combination with other modifications (red boxes). CMA317 displayed no off-target binding to peptides, but did not bind to the H3K9me2 peptide that also harbors K14ac (C22).

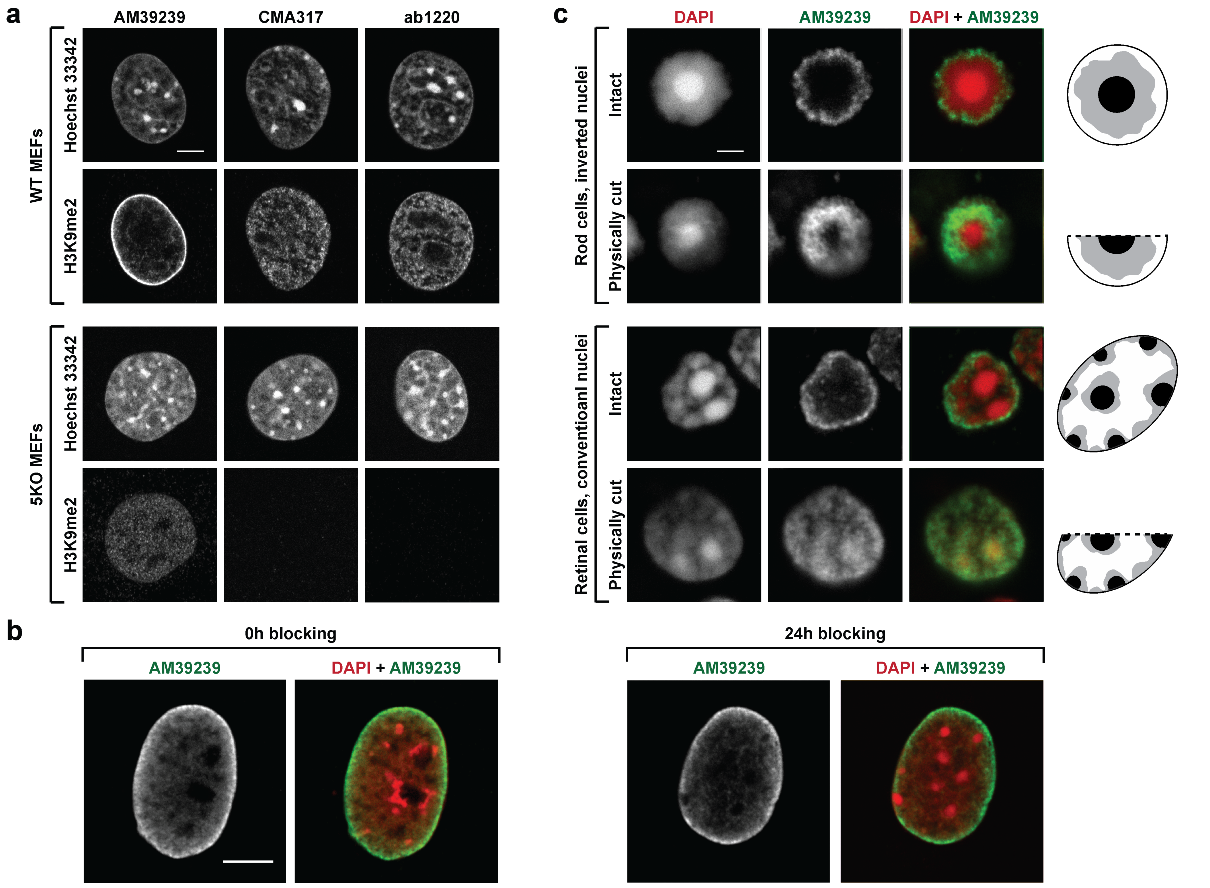

Supplementary Figure S3. Tests for Ab specificity and penetration capacity. a, Immunofluorescence staining with single anti-H3K9me2 Abs in WT (top) and 5KO MEFs nuclei lacking H3K9me2 (bottom). **b**, Immunofluorescence staining with AM39239 in nuclei of myoblasts with and without BSA blocking. **c**, Immunostaining with AM39239 in nuclei of retinal cells fixed in suspension and physically cut within cryosections. Undisturbed rod and non-rod nuclei within the 14 µm thick sections display staining of the peripheral rim typical for the Ab (top layers). Cells at the cryosection surface with physically cut nuclei display chromatin staining throughout the cut surface (bottom layers). All images are single optical sections. Scale bars: 5 µm

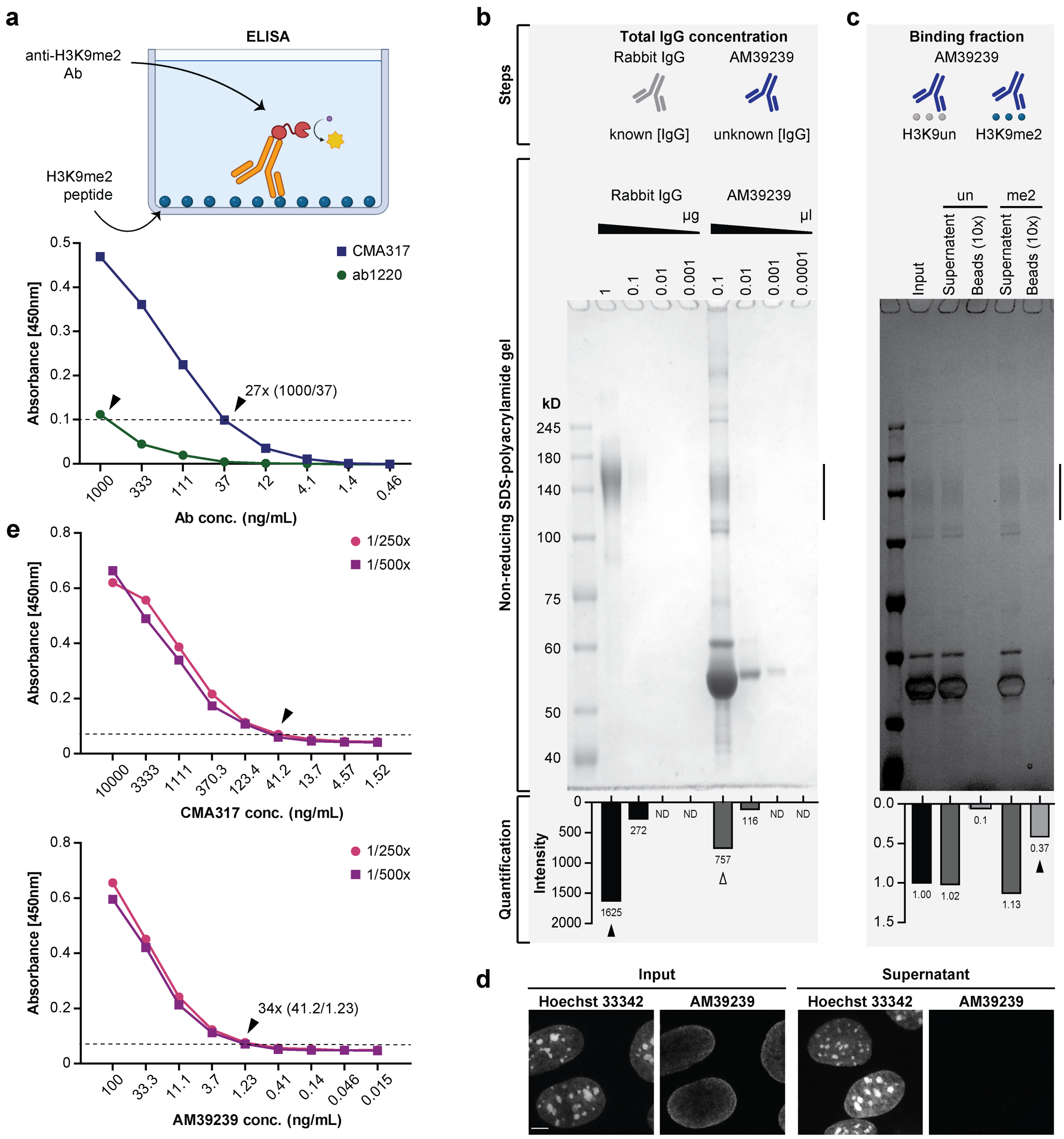

Supplementary Figure S4. Estimating the relative binding affinities of anti-H3K9me2 Abs. a, ELISA profiling of the monoclonal CMA317 and ab1220 Abs comparing the binding affinities to the H3K9me2 epitope. H3K9me2 peptide-coated wells were incubated with a three-fold dilution series of Abs. Similar signals were observed at 27-fold different dilutions between the two Abs (e.g., 1,000 ng/ml CMA317 and 37 ng/ml ab1220) (arrowhead). **b**, Strategy to measure the total concentration of IgG molecules in AM39239 anti-sera. Known amounts of purified rabbit IgG and a serial dilution series of AM39239 were loaded onto a non-reducing SDS-polyacrylamide gel (middle) and the intensities were measured (bottom). 0.1 μl of AM39239 (empty arrowhead) contains ~0.5 μg IgG (arrowhead), indicating an IgG concentration of ~5 mg/ml in AM39239. **c**, Strategy to measure the total H3K9me2 binding fraction of AM39239 anti-sera. AM39239 IgG captured using H3K9 or H3K9me2 beads were loaded onto an SDS-polyacrylamide gel (middle) and the intensities were measured (bottom). The H3K9me2-bound fraction represents ~4% (arrowhead) of total IgG (~0.2 mg/ml) in AM39239. **d**, AM39239 immunofluorescence staining of input (left) and supernatant post-binding to the H3K9me2 beads (right) as represented in c. Lack of signal after binding indicates that most of the specific anti-H3K9me2 IgG molecules were captured by the beads. Scale bar: 5 μm. **e**, Cross-species comparison between mouse CMA317 (top) and rabbit AM39239

(bottom). To ensure comparable detection, peroxidase-conjugated secondary Abs were used at saturated concentrations (1:250, circles and 1:500, squares). Note that the curves for both dilutions overlap, confirming saturation of the detection signal. Comparison of detection thresholds (absorbance = 0.7; arrowheads) at ~1.23 ng/mL for AM39239 and ~41.2 ng/mL for CMA317 reveals an approximately 34-fold higher affinity for AM39239. Schematic in a was created in BioRender. Chudzik, K. (2026) https://BioRender.com/auer958

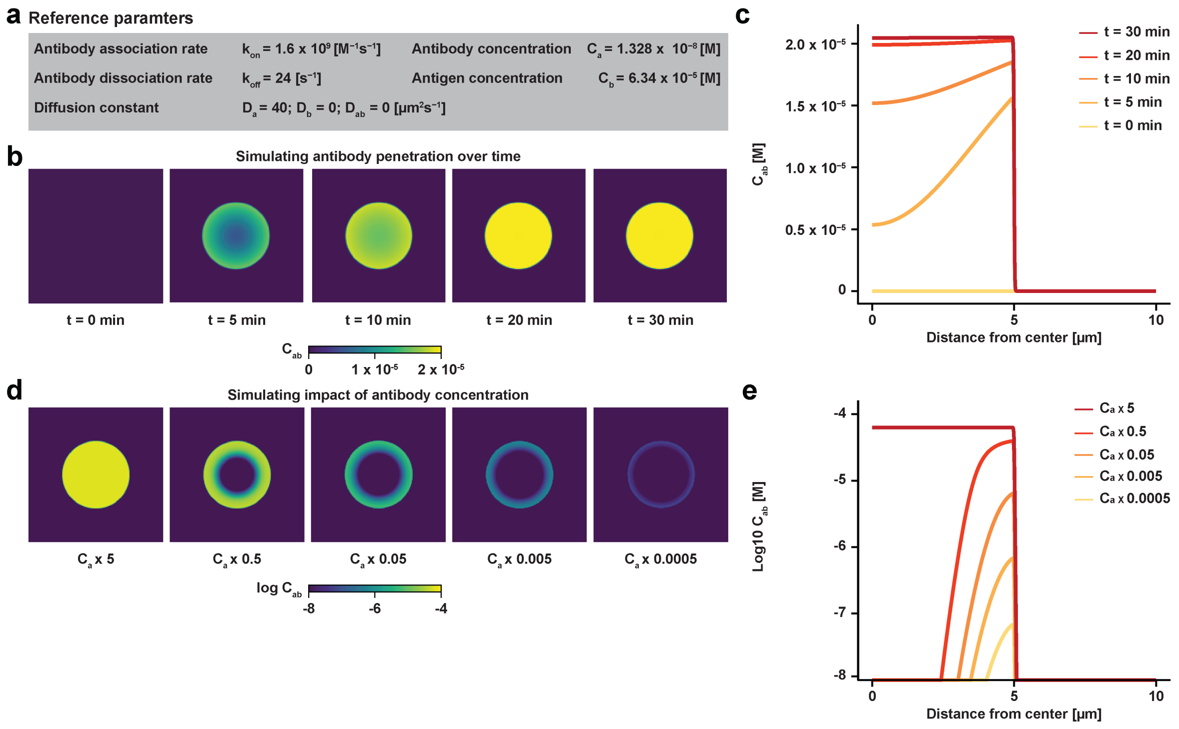

Supplementary Figure S5. Reference parameters and additional perturbations of Ab staining simulations. a, Reference parameters for simulations (66, 72-74). **b**, Time evolution of the concentration field C_{AB} with the reference parameters listed in (a). Over time, a concentration gradient develops and dissipates within approximately 20 min. **c**, Concentration field C_{AB} as a function of the distance from the center of the nucleus over time, as shown in b. Initially, a concentration gradient forms as the antibody diffuses inward and binds to the antigen. Over time, this gradient progressively diminishes as the system reaches equilibrium. **d**, Concentration field C_{AB} (at t=30min) for different initial antibody concentration (C_A) and with a 10-fold increase in k_{on} and 10-fold decrease in k_{off} compared to reference parameters listed in (a). Note that simulations are performed at fixed simulation volume (i.e., assuming cells are diluted to the same volume density). **e**, Concentration field C_{AB} as a function of the distance from the center of the nucleus at 30 min for different initial antibody concentrations, as shown in d. At 5x baseline initial concentration, the concentration at 30 minutes is relatively uniform, whereas at 0.0005x baseline, the concentration remains primarily peripheral.

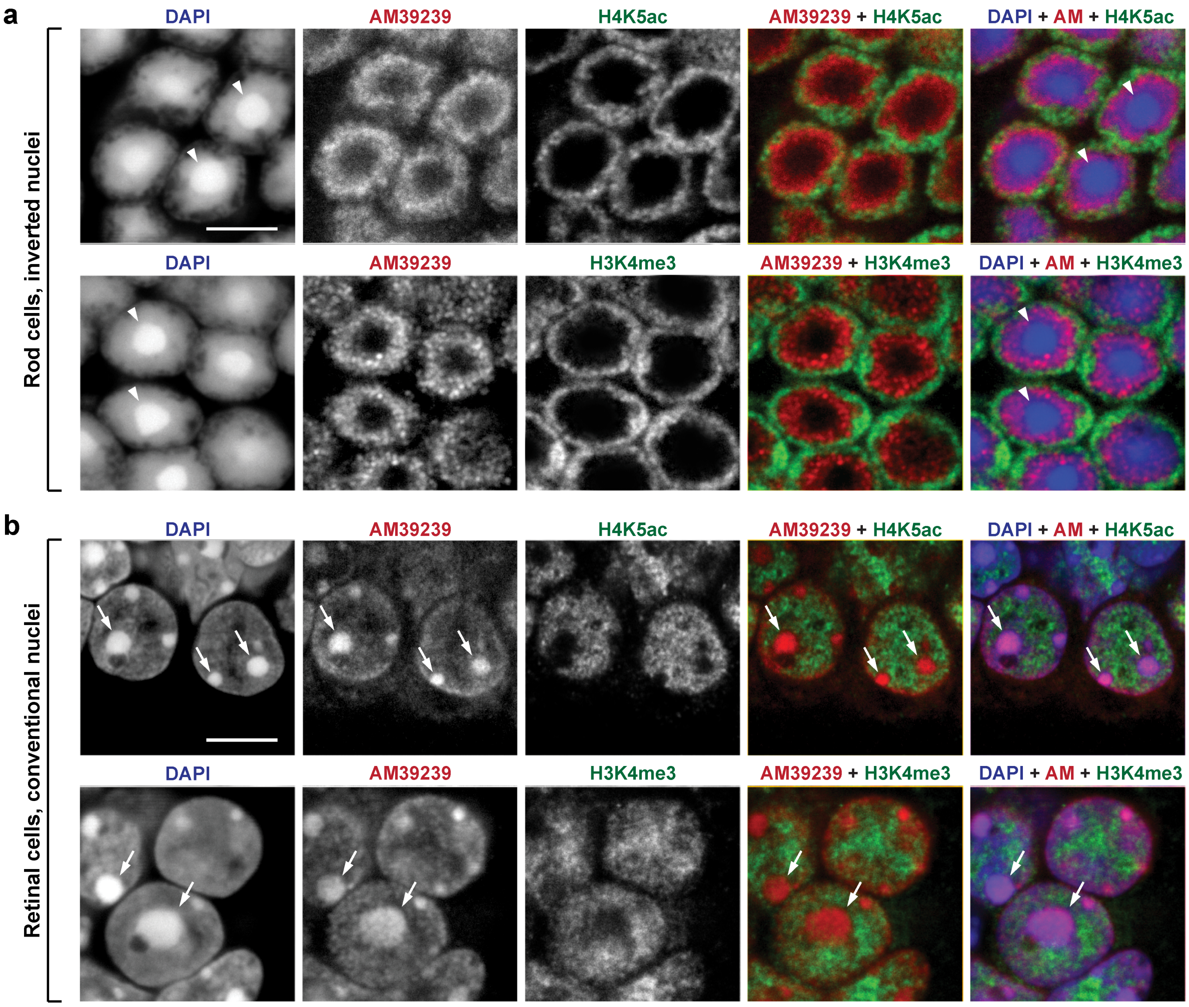

Supplementary Figure S6. Immunostaining of rods (a) and non-rod cells (b) in retinal sections. Pairwise staining with Ab for heterochromatin, AM39239 (red), and Abs for euchromatin, H4K5ac or H3K4me3 (green). In rods (a), AM39239 stains the internal layer of heterochromatin located inwards from the peripheral layer of euchromatin. In non-rod cells (b), staining with AM39239 is mostly excluded from euchromatin and located at the nuclear periphery. Note that chromocenters (blue) in non-rod neurons are stained by the AM39239 Ab (arrow), whereas rod chromocenters are negative (arrowheads). All images are single optical sections. Scale bars: 5 µm.

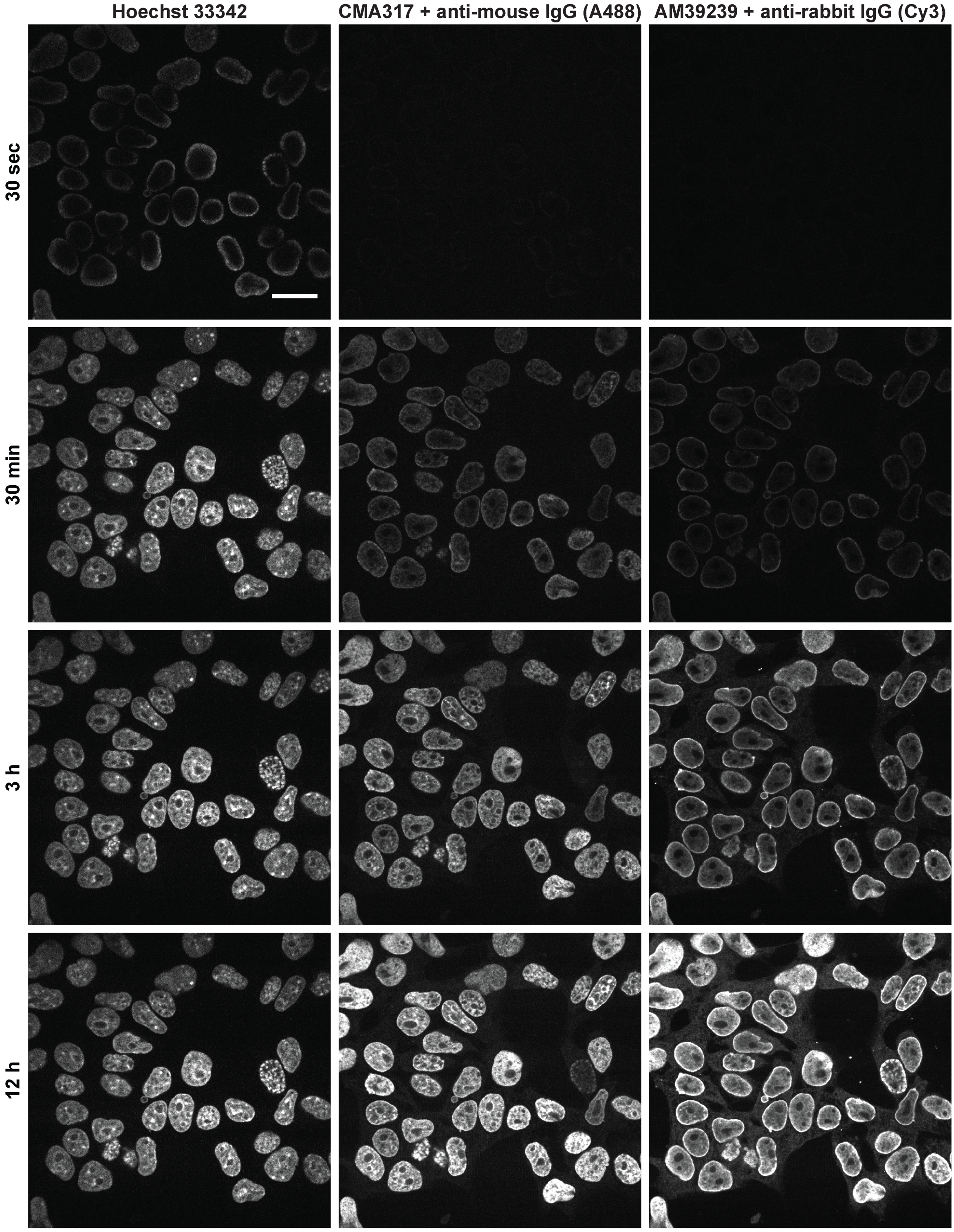

Supplementary Figure S7. Time-lapse imaging of simultaneous immunofluorescence staining with CMA317 and AM39239 Abs. Immunofluorescence staining in nuclei of fixed and permeabilized HeLa cells incubated with CMA317 (preincubated with Alexa Fluor 488-conjugated anti-mouse IgG), AM39239 (preincubated with Cy3-conjugated anti-rabbit IgG), and Hoechst 33342. Staining dynamics were captured with time-lapse imaging for 16.5 hours with 5 min intervals. Note that nuclear rim staining is observed for all probes at early time points. Nuclear interior regions gradually become stained with Hoechst and CMA317, whereas the peripheral staining with AM39239 persists even after 12 h. See Supplemental Video 1 for the full time-series. Scale bar: 20 µm.

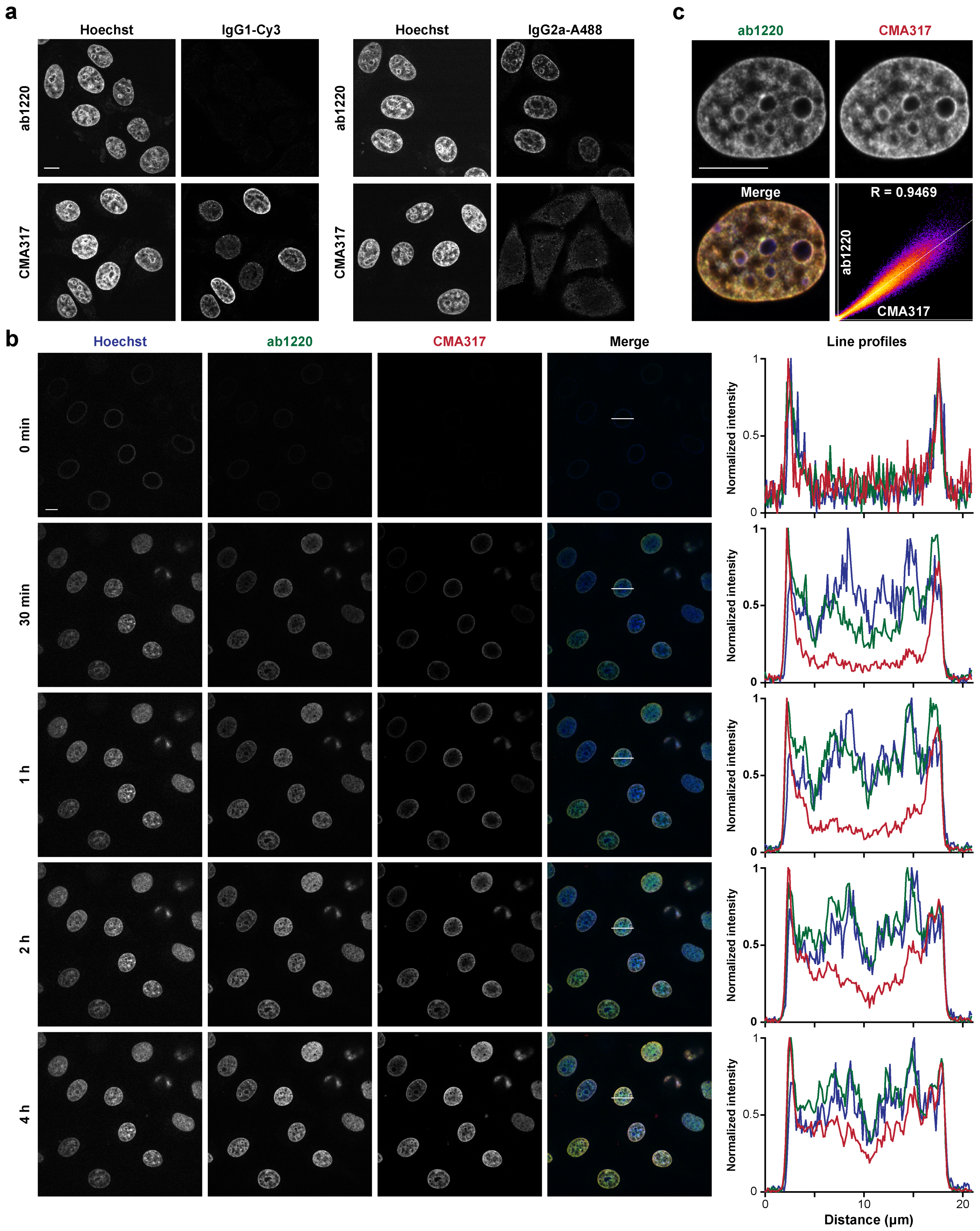

Supplementary Figure S8. Time-lapse imaging of simultaneous immunostaining with CMA317 and ab1220 Abs. Immunofluorescence staining in HeLa nuclei with two mouse anti-H3K9me2 Abs: CMA317 (IgG1 subclass) and ab1220 (IgG2a subclass). **a**, Validation of mouse subclass-specific secondary Abs. **b**, Time-lapse imaging of Ab penetration dynamics over 4 h (left) and corresponding line profile quantifications of signal intensities (right). The white line on images indicates the axis used for line profile analysis. Note that the lower-affinity Ab (ab1220) penetrates the nuclear interior more rapidly than CMA317. See Supplemental Video 2 for the full time-series. **c**, Co-localization of H3K9me2 signal after overnight incubation. Representative nucleus and pixel intensity correlation plot are shown. Pearson correlation

coefficient (R) was calculated for n = 10 nuclei. All images are single optical sections. Scale bars: 10 µm.

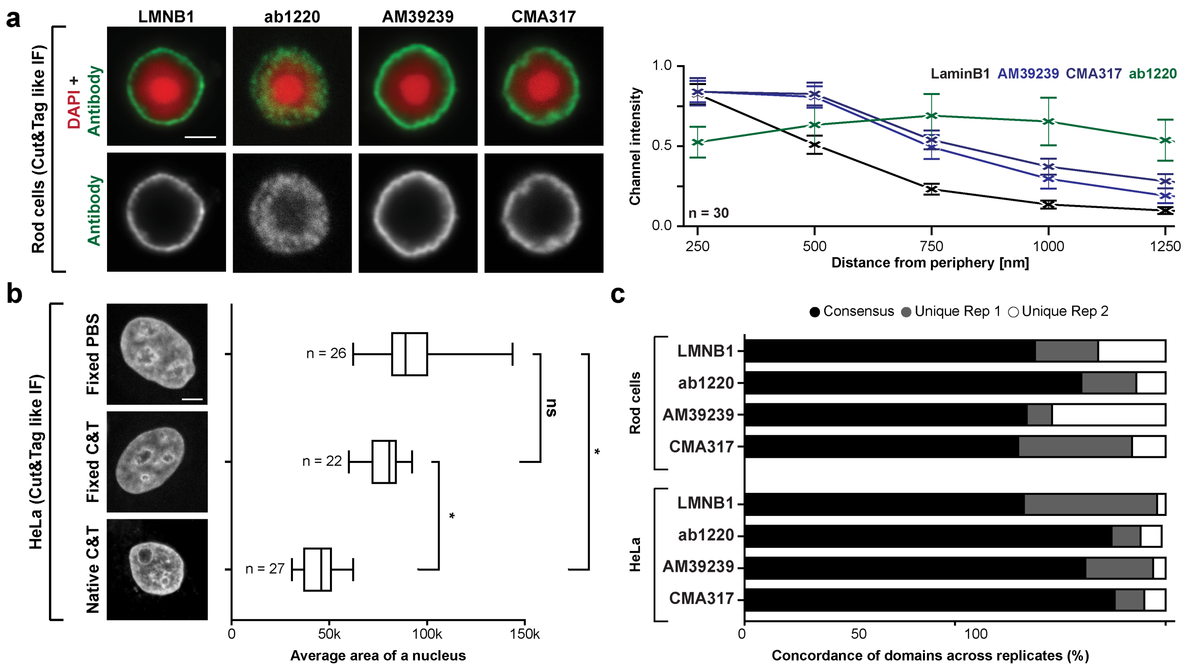

Supplemental Figure S9. Impact of CUT&Tag experimental conditions on nuclear size and Ab signal distribution. a, Immunofluorescence staining with indicated Abs in rod cell nuclei under CUT&Tag conditions (left) and evaluation of the signal radial distribution (right). Note that AM39239 and CMA317 fail to penetrate into the heterochromatic nuclear interior. **b**, DAPI staining in HeLa cell nuclei under the indicated buffer and fixation conditions, with representative example images (left) and quantification of nuclear area (right). Statistical significance (*= p < 0.0001) was determined by an unpaired t-test. ns = not significant. **c**, Fraction of consensus domains (detected in both replicates) and unique domains for indicated Abs in rod cells (top) and HeLa (bottom). All images are single optical sections. Scale bars: a - 2 µm; b - 5 µm.

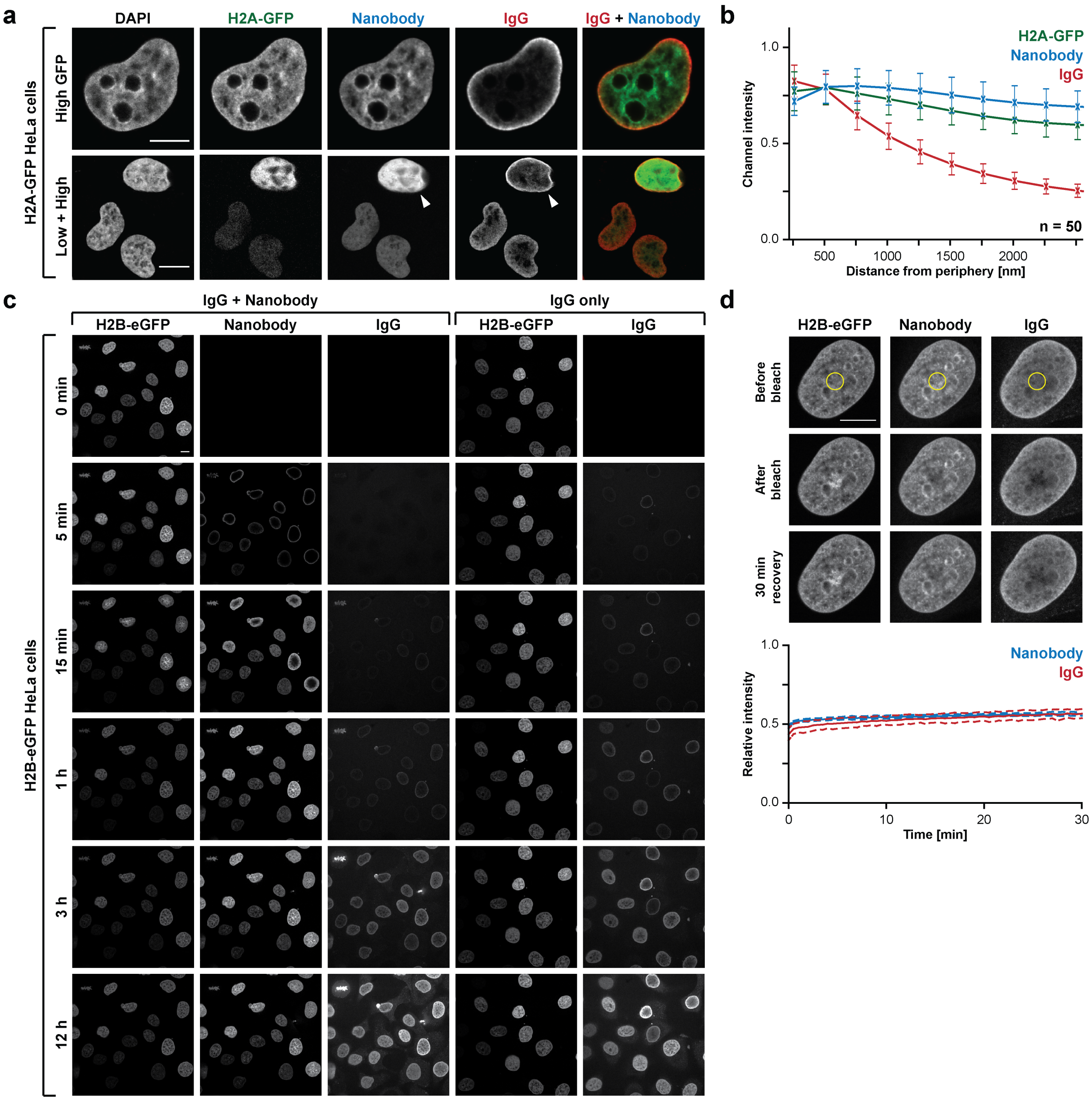

Supplementary Figure S10. Anti-GFP nanobodies are not affected by Ab-trapping. a, Immunostaining with anti-GFP rabbit IgG and nanobody in nuclei of HeLa cells with variable expression of H2A-GFP. Note that the anti-GFP IgG is trapped at the nuclear periphery in cells highly expressing H2A-GFP (arrowhead), while the anti-GFP nanobody stains the whole nucleoplasm. **b**, Quantification of radial signal distributions of indicated Abs. **c,** Immunostaining with anti-GFP rabbit IgG alone (right) or together with a nanobody (left) in nuclei of HeLa cells with variable expression of H2B-eGFP. Staining dynamics were captured with time-lapse imaging for 12 hours with 5 min intervals. **d,** Fluorescence recovery after photobleaching (FRAP) of anti-GFP rabbit IgG and nanobody in nuclei of H2B-eGFP HeLa cells (top). Quantification of signal in the bleached region (yellow circle) over time. All images are single optical sections. Scale bars: 10 µm.

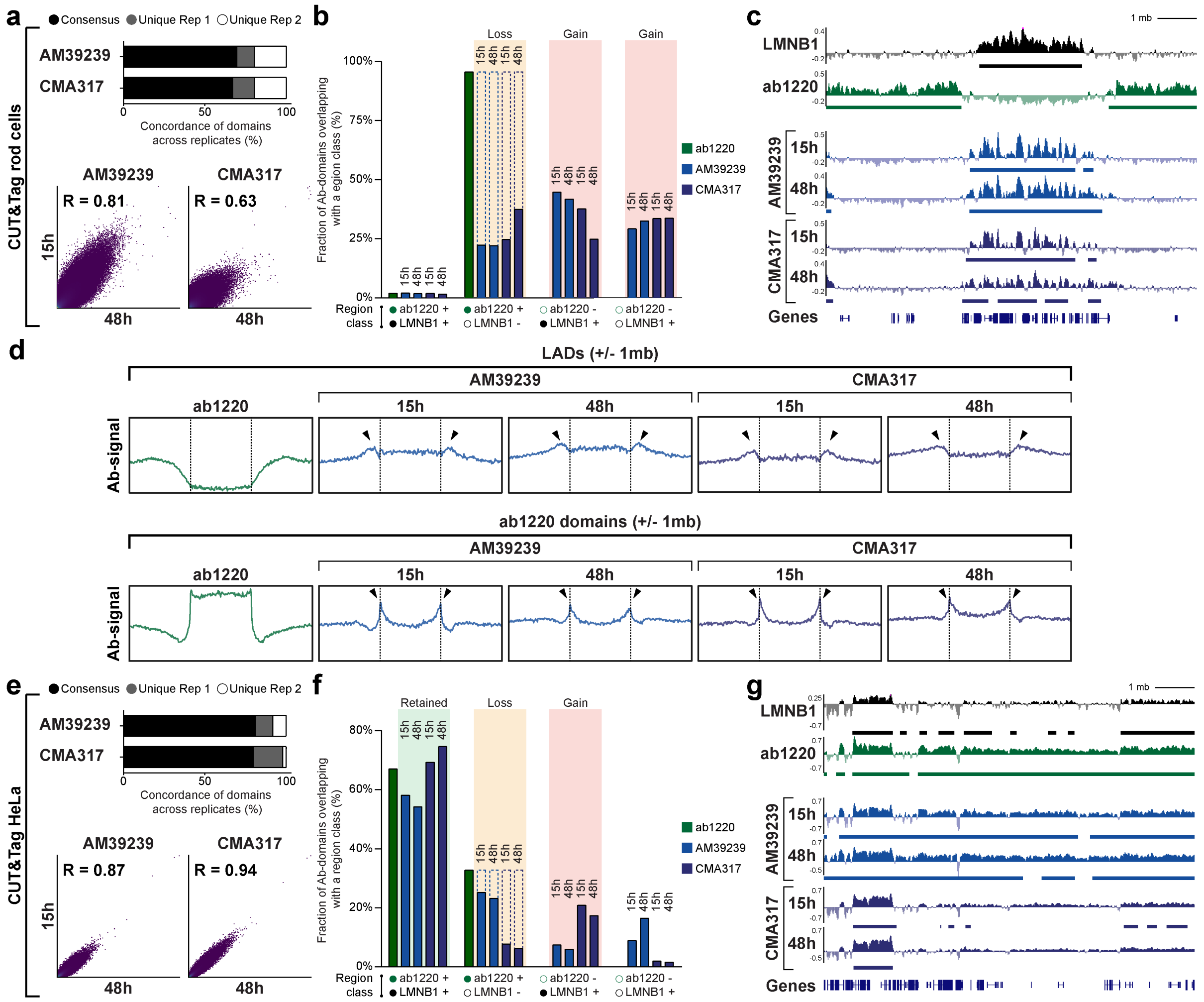

Supplemental Figure S11. Extended primary Ab incubation fails to rescue the Ab-trapping in CUT&Tag experiment. a, Fraction of consensus domains detected in both replicates and unique domains for indicated Abs in rod cells (top). Pearson correlation between 15 h and 48 h incubation timepoints for AM39239 and CMA317 (bottom). **b**, Quantification of the genomic distribution of Ab-defined domains across region classes categorized by the presence or absence of LMNB1 or ab1220 signal. Dashed outlines above the AM39239 and CMA317 bars indicate the ab1220 reference level, highlighting the fraction of missing signal relative to the ground truth. **c**, CUT&Tag tracks in rod cells for the indicated Abs. Tracks represent the average of two biological replicates (log2 signal after IgG control subtraction) with ref-seq genes shown below. Domain calling for each sample is indicated below the corresponding track. **d**, Metaplots showing the distribution of signal for the indicated Abs in rod cells across size-normalized LADs (left) or ab1220 domains (right). 1 Mb of flanking regions are shown in both directions. Arrowheads mark enrichment of AM39239 and CMA317 signal at domain boundaries. **e-g**, Matching quantification and genome browser tracks of CUT&Tag-seq as in a-c, but in HeLa cells.

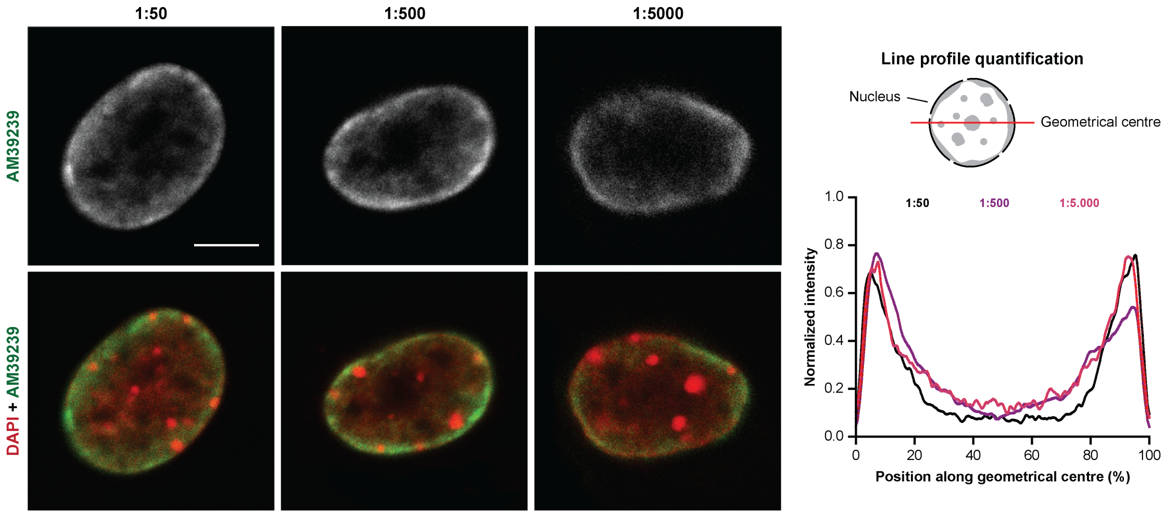

Supplementary Figure S12. Concentration of secondary Abs does not impact antibody-trapping. Immunostaining with AM39239 in myoblasts using varying concentrations of secondary Ab from 1:5000 to 1:50. Representative images (left) and quantification of the signal distribution via line profiles (right) are shown. Schematic of the line profile analysis is depicted above the graph. All images are single optical sections. Scale bar: 5 μm.
